# CSF proteome in multiple sclerosis subtypes related to brain lesion transcriptomes

**DOI:** 10.1038/s41598-021-83591-5

**Published:** 2021-02-18

**Authors:** Maria L. Elkjaer, Arkadiusz Nawrocki, Tim Kacprowski, Pernille Lassen, Anja Hviid Simonsen, Romain Marignier, Tobias Sejbaek, Helle H. Nielsen, Lene Wermuth, Alyaa Yakut Rashid, Peter Høgh, Finn Sellebjerg, Richard Reynolds, Jan Baumbach, Martin R. Larsen, Zsolt Illes

**Affiliations:** 1 Department of Neurology, Odense University Hospital, J.B. Winslowsvej 4, 5000 Odense C, Denmark; 2 Institute of Clinical Research, University of Southern Denmark, Odense, Denmark; 3 Institute of Molecular Medicine, University of Southern Denmark, Odense, Denmark; 4 Department of Biochemistry and Molecular Biology, University of Southern Denmark, Odense, Denmark; 5 Research Group Computational Systems Medicine, Chair of Experimental Bioinformatics, TUM School of Life Sciences Weihenstephan, Technical University of Munich, Munich, Germany; 6 Division Data Science in Biomedicine, Peter L. Reichertz Institute for Medical Informatics of TU Braunschweig and Medical School Hannover, Brunswick, Germany; 7 Danish Dementia Research Centre, Copenhagen University Hospital Rigshospitalet, Copenhagen, Denmark; 8 Service de Neurologie, Sclérose en Plaques, Lyon Neuroscience Research Center, Lyon, France; 9 Department of Neurology, Hospital South West Jutland, University Hospital of Southern Denmark, Esbjerg, Denmark; 10 Regional Dementia Research Centre, Department of Neurology, Zealand University Hospital, Roskilde, Denmark; 11 Department of Clinical Medicine, University of Copenhagen, Copenhagen, Denmark; 12 Danish Multiple Sclerosis Center, Department of Neurology, Copenhagen University Hospital – Rigshospitalet, Glostrup, Denmark., Copenhagen, Denmark; 13 Department of Brain Sciences, Imperial College, London, UK; 14 Department of Mathematics and Computer Science, University of Southern Denmark, Odense, Denmark; 15 Chair of Experimental Bioinformatics, TUM School of Life Sciences Weihenstephan, Technical University of Munich, Munich, Germany

**Keywords:** Immunology, Neuroscience, Molecular medicine, Neurology

## Abstract

To identify markers in the CSF of multiple sclerosis (MS) subtypes, we used a two-step proteomic approach: (i) Discovery proteomics compared 169 pooled CSF from MS subtypes and inflammatory/degenerative CNS diseases (NMO spectrum and Alzheimer disease) and healthy controls. (ii) Next, 299 proteins selected by comprehensive statistics were quantified in 170 individual CSF samples. (iii) Genes of the identified proteins were also screened among transcripts in 73 MS brain lesions compared to 25 control brains. F-test based feature selection resulted in 8 proteins differentiating the MS subtypes, and secondary progressive (SP)MS was the most different also from controls. Genes of 7 out these 8 proteins were present in MS brain lesions: *GOLM* was significantly differentially expressed in active, chronic active, inactive and remyelinating lesions, *FRZB* in active and chronic active lesions, and *SELENBP1* in inactive lesions. Volcano maps of normalized proteins in the different disease groups also indicated the highest amount of altered proteins in SPMS. Apolipoprotein C-I, apolipoprotein A-II, augurin, receptor-type tyrosine-protein phosphatase gamma, and trypsin-1 were upregulated in the CSF of MS subtypes compared to controls. This CSF profile and associated brain lesion spectrum highlight non-inflammatory mechanisms in differentiating CNS diseases and MS subtypes and the uniqueness of SPMS.

## Introduction

Identification of specific molecular markers that reflect the pathology and disease course of multiple sclerosis (MS) is difficult because of the dynamic and complex molecular pathogenesis. Early in the course, MS is characterized by clinically active and silent phases (relapsing–remitting, RRMS). A secondary progressive phase (SPMS) evolves in a subset of patients, where a combination of neurodegenerative processes, adaptive and innate immune responses contributes to the advancing disability, and limits the efficacy of treatments that target mainly systemic adaptive immune responses^1–4^. One out of eight MS patients are diagnosed with primary progressive (PP)MS characterized by the absence of clinical relapses and gradual worsening of disability from onset. Axonal degeneration, cortical lesions, innate immune responses by resident cells, inflammatory demyelination, and remyelination significantly influence the prognosis and long-term outcome of MS^1,4,5^. Early prediction of mechanisms that culminate in the progressive phase may provide a more individualized treatment approach and postpone the secondary phase^6^.

Hypothesis-generating exploratory omics are effective tools for revealing novel molecular pathways and quantifying differentially expressed molecules to identify multiple markers that may predict disease outcomes. Mass spectrometry is an analytical technique for the characterization of biological samples and is increasingly used in omics studies as both a nontargeted and targeted approach for discovery proteomics and quantification with high throughput abilities. Proteomics of the cerebrospinal fluid (CSF) reflects more specific changes related to CNS damage than serum, and is a powerful tool for elucidating mechanisms by networks, pathways, protein groups and individual proteins that reflect both the similar and the unique molecular events as inflammation, degeneration, reparation or oxidative stress conditions in the MS subgroups^7^. Multi-omics, i.e. combination of different omics approaches to examine differences and overlap at multiple molecular layers, compartments and species are emerging and may provide better understanding of MS pathophysiology^8,9^.

**Figure 1:**
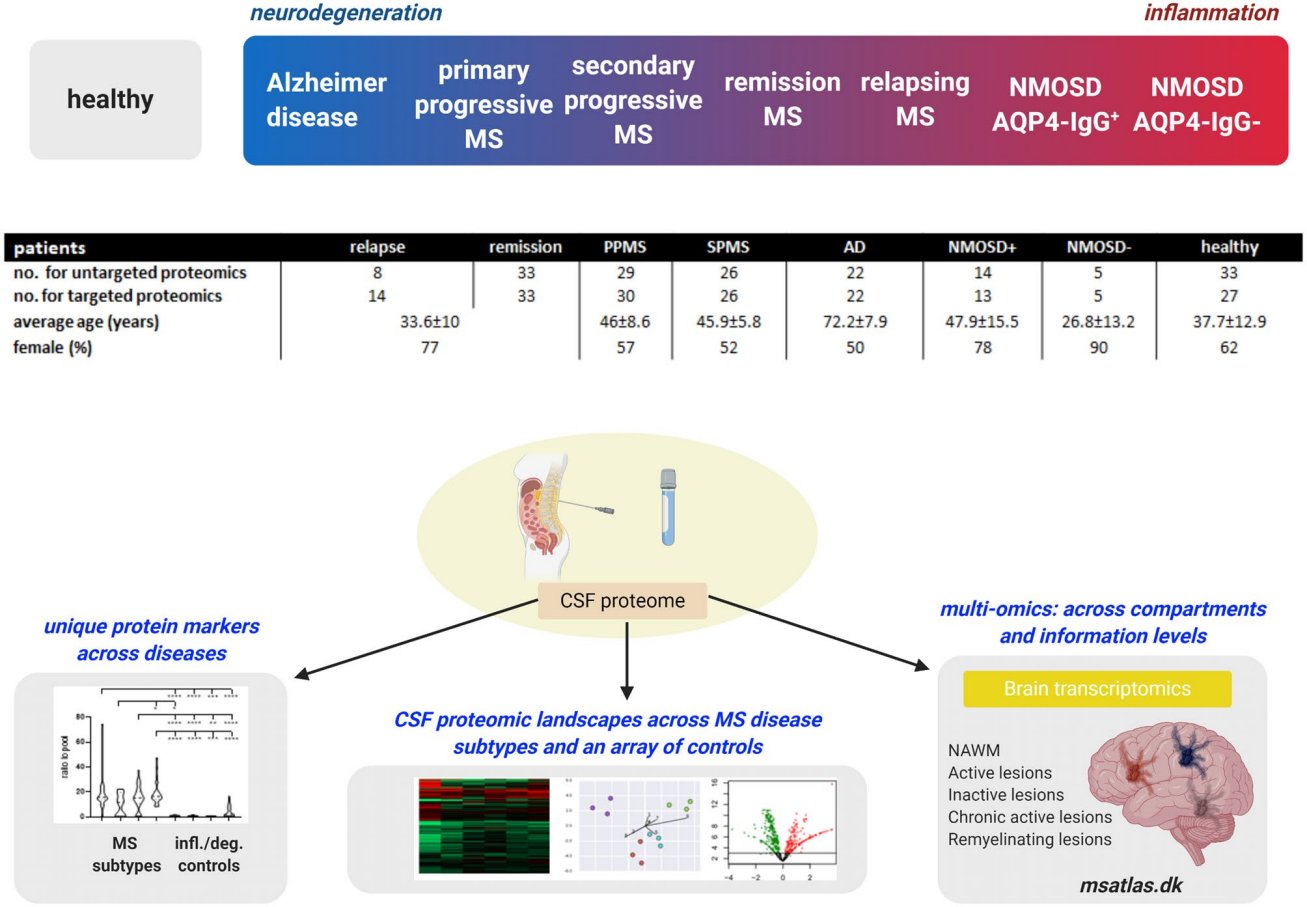
A multi-omics approach to identify molecules that characterize MS subtypes. An outline of the study is shown with an overview of CSF and brain samples collected for the comprehensive proteomic study and its overlap with brain lesion transcriptomes. *MS* multiple sclerosis, *NAWM* normal-appearing white matter, *NMOSD* neuromyelitis spectrum disorder. Created with BioRender.com.

Here, we used a comprehensive two-stage approach, with an untargeted and then a quantitative targeted method to characterize the molecular landscape of the CSF in different phases of MS. We also examined the genes of identified molecules among transcripts in different lesion types in the MS brain^10^. Disease controls were selected to include conditions with strong inflammatory alterations in the CNS without major degenerative processes but with similarity to MS, i.e. neuromyelitis optica spectrum disease (NMOSD) with or without pathogenic antibodies against aquaporin-4 (AQP4-IgG^+^ and AQP4-IgG^−^)^11^, and neurodegenerative conditions associated with innate inflammatory responses in the CNS, i.e. Alzheimer disease (AD)^12^. Our previous study indicated differences in the urine proteome when MS was compared to NMOSD^13^. Based on the different protein abundances in 169 CSF samples, we: (i) clustered the diseases and MS subtypes to create the CSF proteomic landscapes across MS subtypes and an array of controls; (ii) selected 299 proteins that were quantified in 170 individual CSF of the MS subgroups and controls to identify novel unique protein markers across diseases; and (iii) linked the unique CSF proteins with MS brain lesion transcriptome signatures using multi-omics comparison across compartments and information levels^10^ (Fig. 1).

## Materials and methods

### Study design and participants

We examined the CSF proteome in a two-stage approach, with an untargeted (n = 169) and then a quantitative targeted method (n = 170) (Supplementary Fig. S1). The same CSF samples were used for both untargeted and targeted proteomics, except that a few additional samples were added for the relapse cohort in the targeted analysis, while the targeted datasets of healthy controls and NMOSD contained less samples (Fig. 1).

CSF samples were obtained through regional, national and international collaboration (Denmark, France, Hungary) from patients with newly diagnosed, untreated RRMS (age 33.6 ± 10 years, 77% female) in relapse (n = 14) or remission MS (n = 33), untreated PPMS (n = 30, age 49 ± 8.6, 57% female), untreated SPMS (n = 26, age 45.9 ± 5.8 years, 52% female), AD (n = 22, age 72.2 ± 7.9 years, 50% females), NMOSD AQP4-IgG^+^ (n = 14, age 47.9 ± 15.3 years, 78% female), NMOSD AQP4-IgG^-^ (n = 5, age 26.8 ± 13.2, 90% female) and healthy controls (n = 33, age 37.7 ± 12.9 years, 62% female). None of the patients with MS had disease-modifying therapy. Relapse was verified by neurologists, and samples were taken within maximum a month after the first relapse symptoms. Patients with AQP4-IgG^−^ NMOSD were not treated with immunosuppressive medications, while patients with AQP4-IgG^+^ NMOSD received azathioprine or mycophenolate mofetil. NMOSD was stable in all patients.

CSF samples were obtained by lumbar puncture, collected in polypropylene tubes and gently mixed. The samples were centrifuged at 2000×*g* for 10 min at 4 °C to remove cells and other insoluble materials and stored in polypropylene tubes at − 80 °C pending analysis.

The study was conducted in accordance with the approval of the Danish National Ethics Committee (S-20120066), and informed consent was obtained from each participant.

### Sample preparation for proteomic discovery

CSF samples of each disease group were pooled into one of three sample pools producing three technical replicates (Supplementary Fig. S1a). Proteins were ethanol/acetone precipitated, re-dissolved in 7M urea, 2 M thiourea, 20 mM dithiothreitol (DTT), and the protein amount was estimated using Qubit Protein Assay (Thermo Fisher Scientific). Following alkylation, pH of the samples was adjusted to 8 and proteins were digested with LysC (0.02 AU/mg proteins) for 4 h, and then with trypsin (50:1 ratio) overnight at 37 °C. Peptides were reversed phase (RP) purified using homemade columns of C8/R2 and C18/R3 (Applied BiosystemsTM). Purified peptides were re-dissolved in 0.1% formic acid. The peptide amount in each sample was determined by amino acid composition analysis (AAA). Subsequently, equal amounts of each sample pool were labelled with one of the iTRAQ 8plex reagent labels according to manufacturer protocol. The bulk peptide sample was fractionated using hydrophilic interaction chromatography (HILIC), and each fraction was further separated by reversed phase chromatography prior to identification by mass spectrometry (Q Exactive HF, Thermo Fisher Scientific). The three technical replicates of the sample pools were run separately (Supplementary Fig. S2a).

### Statistical analyses for selection of proteins

Proteome Discoverer software (further PD software, Thermo Scientific, v1.4) was used to process the raw mass spectrometry (MS) files, identify the proteins and generate quantitative data which was further processed by three parallel approaches.

#### ANOVA-based (analysis of variance)

For each peptide, ANOVA was performed with the lmPerm R package to determine difference between groups. Afterwards, to determine which pairs of groups showed most differences, the Tukey's HSD (honest significant difference) test was performed as post-hoc analysis.

#### Limma-based (linear models)

Linear regression and analysis of variance were performed with the limma R package. The ratios of a specific protein between two compared groups were log_2_ transformed, normalized to the median, and the 3 replicates merged into one, and proteins were significant according to q-values (FDR < 0.1). The resulting data were visualized in volcano plots and heatmaps using Perseus^14^.

#### Complementary analysis of the three replicates

Using the PD software, for each of the three sets the coefficient of variation CV of proteins (any subject group to healthy subjects) within the set as well as the ratio of the mean abundance between the sets were calculated. A protein was selected for further analyses, if the ratio was larger (or smaller) than 1 + 2xCV (or reciprocal). Subsequently, the PD software calculated a “global” ratio for a protein based on data from the three sets compared to healthy samples (and CV within the combined sets). Proteins were finally selected, if the protein expression was larger (or smaller) than 1 + 2xCV (or reciprocal) at least between two different conditions, and was consistently altered in a minimum of two of the three sets.

### Linear discriminate analysis (LDA)

To reduce any possible batch effect, the three pools were merged after scaling them individually (per protein). An F-test based feature selection was performed, where only proteins with a FDR < 0.05 (ANOVA) were considered. Next, the set of candidate proteins were pruned for collinearity by iteratively removing the protein with the highest variance inflation factor (VIF), until only proteins with VIF < 10 remained. This resulted in 11 proteins, which were used to conduct a linear discriminant analysis (LDA). Additionally, the test was also performed only on the MS samples resulting in 8 proteins responsible for the subgroup separation according to the LDA.

### Pathway analysis

After the data were normalized to control samples, Ingenuity Pathway Analysis (IPA) was used to identify molecular pathways and perform functional analysis between different disease groups and subgroups.

### Sample preparation for quantification

CSF from each patient was precipitated with ethanol/acetone, dissolved in urea buffer containing DTT, as described in a previous paper with parallel reaction monitoring (PRM)^8^. Total protein content was estimated by AAA, and 10 μg of proteins were digested with trypsin. After digestion, Stable Isotope Standards (SIS) mix was added in equal volume to every sample (both previously prepared^8^ and additional ones). Peptides in each sample were labelled with one of the TMT 11plex label. A pooled sample was prepared by mixing a small amount from approximately half of all the available samples. This pooled sample was labelled with TMT 11plex 126 label. Subsequently, this pooled sample was split equally into 17 samples, and mixed with ten other patient samples in a random manner (Supplementary Fig. S2b). There were 17 TMT sets each containing at least one (if available) sample from every patient group. Samples were randomized so that each set contained a representative of each patient group, and each sample of every patient group was labelled with a different TMT label.

Peptides of every set were fractionated by HILIC and analyzed by liquid chromatography tandem mass spectrometry (LC–MS/MS). The LC method total runtime varied in length between 67 to 143 min depending on signal intensity of the HILIC fractions. Most of the peptides separated during the linear increase of solvent B from 10 to 35% in 38 to 120 min (corresponding to the total runtime). MS settings: Full MS: Resolution at 120,000, AGC target 3e6, Maximum IT 100 ms, scan range: 325–1600 *m/z*. MSMS settings: Resolution at 60,000, AGC target 1e5, Maximum IT 100 ms, isolation window 1.2 *m/z*, NCE: 32, top 15 most intense ions of 2–4 charges (positive mode), dynamic exclusion of 15–20 s.

### Data processing and statistical analyses of validated proteins

The raw data was processed with the ProteomeDiscoverer software (v2.3). The samples used for analysis contained SIS standard added in the same amount to each sample and labelled with TMT along with all the other CSF peptides. Each patient group was set as one of the Categorical factors, and every patient within a patient group was set as a Biological replicate. The Pool sample was set as “Control” and every patient sample was set as “Sample”. The scaling parameter was set “On Average Control”. In this way, samples were normalized and scaled to the Pool (which is a common/identical sample across the 17 replicates). The software calculated ratios for protein abundances between any patient group and healthy controls based on proteins identified and quantified in corresponding samples from all the 17 Sets. In an alternative approach, the quantitative data from ProteomeDiscoverer were extracted and further processed in Excel (Microsoft). The constant ratio of CSF proteins to SIS were used to calculate normalization factors within each of the 17 TMT sets. Additionally, this SIS normalization could also be used for correcting the few samples that contained less than 10 μg of proteins and different amount of volume. After normalization, an average ratio for each protein (for every patient group) was calculated based on the ratios to the corresponding protein in the Pooled sample. The significance of the ratios was validated by ANOVA by using PRISM and PolySTest software^15^.

### Human brain lesion signature

We recently characterized and microdissected 73 lesions from brain of 10 patients progressive MS covering different lesion types: normal-appearing white matter (NAWM), active, chronic active, inactive, and repairing lesions; and as controls, 25 white matter (WM) areas from five brains without neurological disease^10,16^. Paired-end next-generation-sequencing was performed on the total RNA followed by data processing, alignment and statistical analyses^10,16^. The comprehensive transcriptome data was used to create an online web-tool (www.msatlas.dk) to explore RNA profiles in lesion evolution of progressive MS. Gene names of the protein of interest from the proteome data were uploaded on the msatlas.dk, and heatmaps were produced of genes present in the human MS brain^10^. Stars were added, when there was a significant difference (FDR < 0.05) between MS lesion type and control WM from non-neurological disease brain areas.

### Immunohistochemistry and RNAscope of chronic active brain lesion

Human postmortem brain tissue were supplied by the UK Multiple Sclerosis Tissue Bank (UK Multicentre Research Ethics Committee, MREC/02/2/39), funded by the Multiple Sclerosis Society of Great Britain and Northern Ireland (registered charity 207,495). Fresh-frozen blocks containing chronic active lesion from progressive MS patients were sectioned (10-μm), PFA-fixed, blocked in PBS with 10% normal horse serum (NHS) and immunostained with rabbit CHI3L1 (monoclonal antibody) 1:200 (Abcam) followed by biotinylated secondary antibody (Jackson Immunoresearch Laboratories, Cambridgeshire, UK), avidin/biotin staining (Vector Laboratories, Burlingame, CA) and 3,3′-diaminobenzidine (DAB) staining (Vector Laboratories, Burlingame, CA). The RNAscope 2.5 Duplex Assay (ACD Biosystems) was performed according to the ACD protocol for fresh-frozen tissue. Chronic active lesions were hybridized with two mRNA probes per experiment. Hs-GFAP (Cat No. 311801) was used as the astrocyte marker together with Hs-CHI3L1 (Cat No. 408121). The probes were amplified according to manufacturer’s instructions and labeled with the following red or green color for each experiment. The Hs-CHI3L1 probe was also combined with immunohistochemistry (anti-GFAP and anti-MHCII, Abcam) as described above.

## Results

### Global CSF proteome landscape of MS subtypes: untargeted analysis of the CSF proteome in MS subgroups and controls

**Figure 2:**
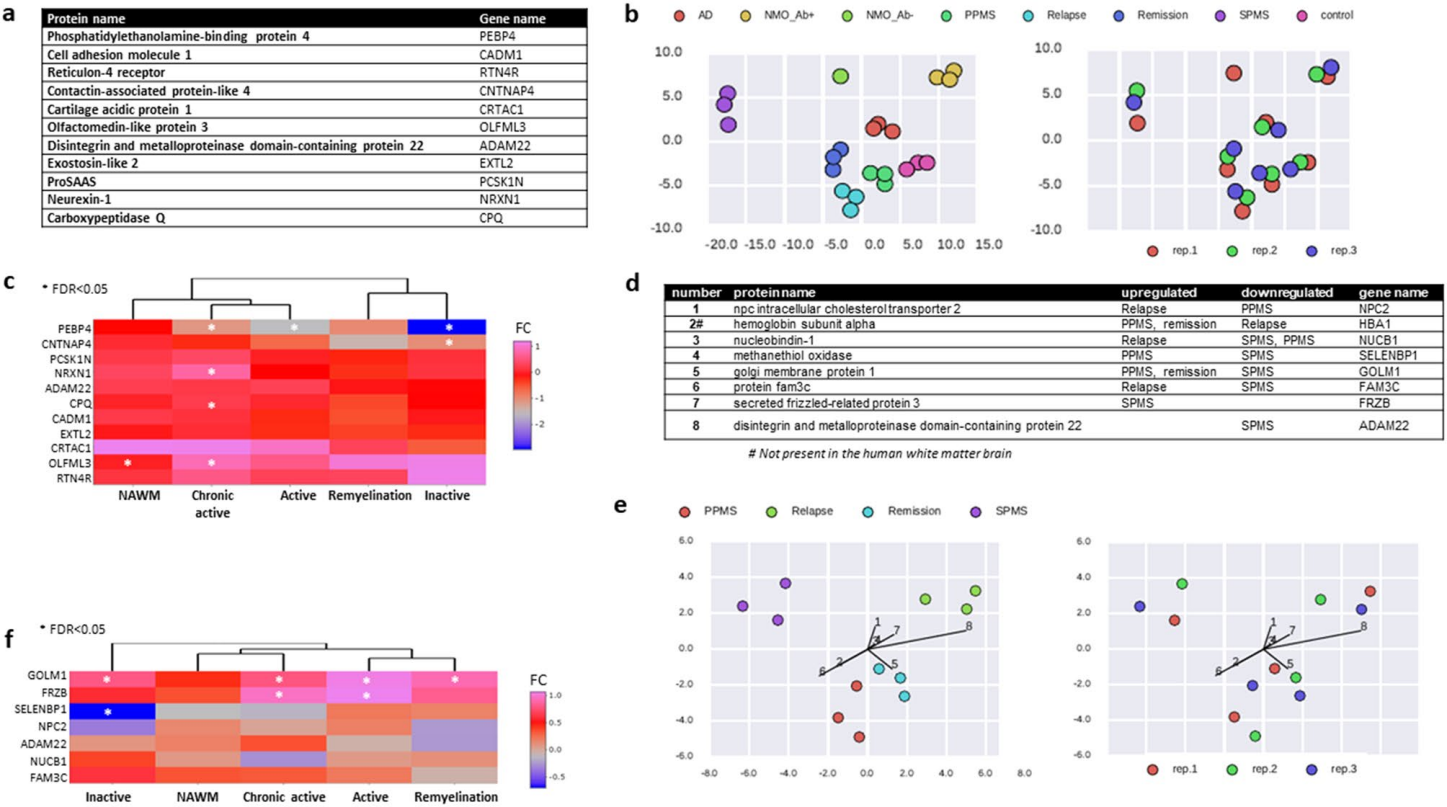
Protein combinations discriminating CNS diseases and MS subtypes. (**a**) Combination of the 11 proteins listed were able to discriminate PPMS, MS in relapse, MS in remission, SPMS, AD, AQP4-IgG^+^ NMOSD, AQP4-IgG^−^ NMOSD, and healthy controls. (**b**) Disease-specific discrimination by using linear discriminant analysis (LDA). (**c**) Hierarchical clustering of expression of genes encoding the 11 proteins in different lesions in the MS brain white matter. Stars represent significantly differentially expressed genes (FDR < 0.05) compared to non-neurological disease brains. Colour represents the log_2_ fold change (FC). (**d**) Combination of the 8 proteins listed were able to discriminate among MS subtypes. (**e**) LDA classifier showing discrimination between MS patients in relapse, remission, and with PPMS and SPMS based on the 8 proteins. The "connecting threads" in the graph show the contribution of the 8 proteins used for the LDA. (**f**) Hierarchical clustering of brain lesion expression of genes encoding the 8 compound proteins that differentiate among MS subtypes. Stars represent significantly differentially expressed genes (FDR < 0.05) compared to non-neurological disease brains. Colour represents the log_2_ fold change (FC). *CSF* cerebrospinal fluid, *PP/SPMS* primary/secondary progressive multiple sclerosis, *AD* Alzheimer disease, *NMO Ab*^*+/−*^ neuromyelitis optica spectrum disorder positive/negative for aquaporin-4 antibody, *FDR* False discovery rate.

Altogether, we detected 878 proteins in the 169 CSF samples. By using F-test based feature selection, 11 proteins were able to distinguish the disease (sub)groups (Fig. 2a). These 11 proteins were used to conduct a linear discriminant analysis (LDA) that focuses on maximizing the separability among the known disease groups and healthy controls: there was no overlap between the different disease groups, and no influence of the technical batch effect (Fig. 2b). NMOSD (AQP4-IgG^−^ and AQP4-IgG^+^) and SPMS were the most distinct groups both from each other and from healthy controls, PPMS, RRMS (relapse, remission) and AD. The presence of genes coding these 11 proteins in the MS brain was examined by using www.msatlas.dk. All were expressed in the MS brain, and 5 of them were significantly differentially expressed in different lesion types especially in the chronic active lesion type (*PEBP4, CNTNAP4, NRXN1, CPQ, OLFML3*) (Fig. 2c).

F-test based feature selection was also applied to the MS CSF samples separately and resulted in 8 proteins differentiating the MS subtypes (early MS in remission and relapse, SP and PPMS) (Fig. 2d). The LDA according to these 8 proteins identified also the SPMS subtype as being the most different (Fig. 2e). Seven of the 8 genes encoding for the proteins were present in the MS brain, and 3 were significantly differentially expressed: *GOLM* in all the lesion types (active, chronic active, inactive and remyelinating), *FRZB* in active and chronic active lesions, and *SELENBP1* in inactive lesions (Fig. 2f).

**Figure 3:**
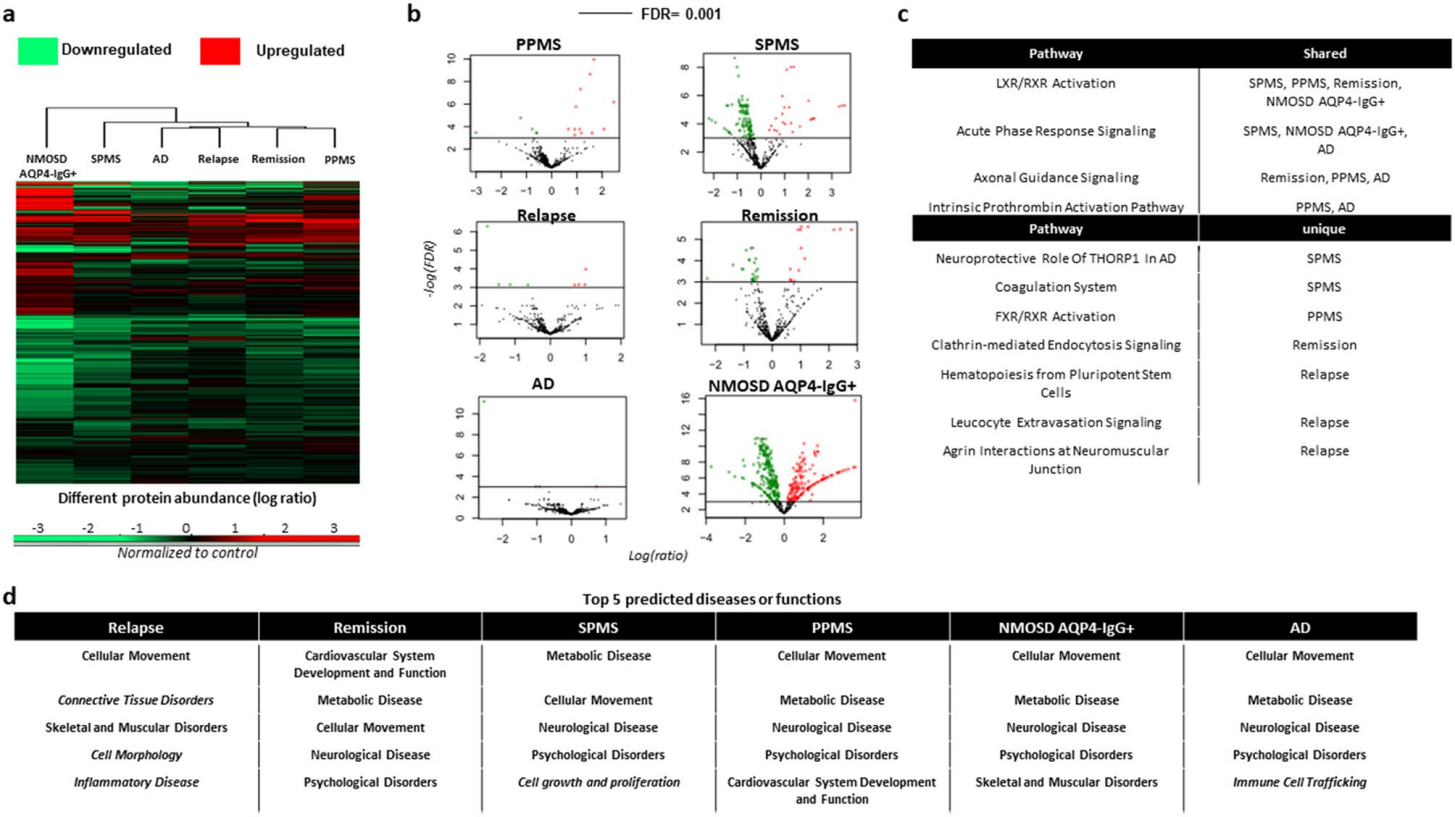
Functional analyses of the CSF proteome of disease groups normalized to healthy controls. (**a**) Heatmap clustering of PPMS, MS in relapse, MS in remission, SPMS, AD, and AQP4-IgG^+^ NMOSD versus healthy control based on the different abundance of the protein levels. Red colour represents upregulation, while green represents downregulation in disease groups compared to healthy control. (**b**) Volcano plots of differentially expressed proteins in each disease group compared to healthy controls. Each point represents the average value of one protein in three replicate experiments. The dark horizontal line is set when the protein expression difference is significant with FDR < 0.001. (**c**) The top scoring canonical pathways for the different disease groups using Ingenuity Pathway Analysis (IPA) and z-score algorithms. (**d**) The top 5 predicted biological functions for each of the disease group compared to healthy control. Italic indicates a unique function for a disease group. *CSF* cerebrospinal fluid, *PP/SPMS* primary/secondary progressive multiple sclerosis, *AD* Alzheimer disease, *AQP4-IgG*^*+*^* NMOSD* neuromyelitis optica spectrum disorder serum positive for aquaporin-4 antibody, *FDR* False discovery rate.

Next, we normalized the protein levels to healthy controls, and the diseases were clustered based on the abundance in protein log_2_ fold change (except AQP4-IgG^-^ NMOSD due to lack of technical replicates) (Fig. 3a, Supplementary Table S1). AQP4-IgG^+^ NMOSD was the most different from the other diseases, and SPMS the most different from the other MS subtypes (Fig. 3a). Volcano maps of normalized proteins in different disease groups also indicated that AQP4-IgG^+^ NMOSD and SPMS had the highest amount of altered proteins compared to healthy controls (FDR < 0.001) (Fig. 3b). For detailed inspection of each protein, and their FDR and log ratio please see Supplementary Table S1. Functional classification and molecular pathways of the proteome in the different diseases were generated by Ingenuity Pathway Analysis (IPA) (Fig. 3c, Supplementary Fig. S3). The most shared pathway was “LXR/RXR Activation” by SPMS, PPMS, MS remission, and AQP4-IgG^+^ NMOSD. “Acute Phase Response Signalling” was shared between SPMS, AQP4-IgG^+^ NMOSD and AD. “Axonal Guidance Signalling” was shared between MS remission, PPMS and AD. PPMS and AD shared “Intrinsic Prothrombin Activation Pathway”. AD and AQP4-IgG^+^ NMOSD shared “Complement”. SPMS had two unique pathways: “Neuroprotective Role Of THORP1 In AD” and “Coagulation System”, while PPMS and remission had one each, “FXR/RXR Activation” and “Clathrin-mediated Endocytosis Signalling”, respectively.

While CSF in MS relapse did not share common top pathways, the top pathways were “Hematopoiesis from Pluripotent Stem Cells”, “Leucocyte Extravasation Signalling” and “Agrin Interactions at Neuromuscular Junction” (Fig. 3c). The distinct biological functional enrichment of MS relapse was also reflected by the top 5 predefined diseases or functions (Fig. 3d). The top network assigned for all the disease groups were “Metabolic Disease”, “Cellular Movement”, ”Neurological Disease” and “Psychological Disorders”, while relapse only shared “Cellular Movement”.

### Unique CSF proteins in disease subtypes

**Figure 4:**
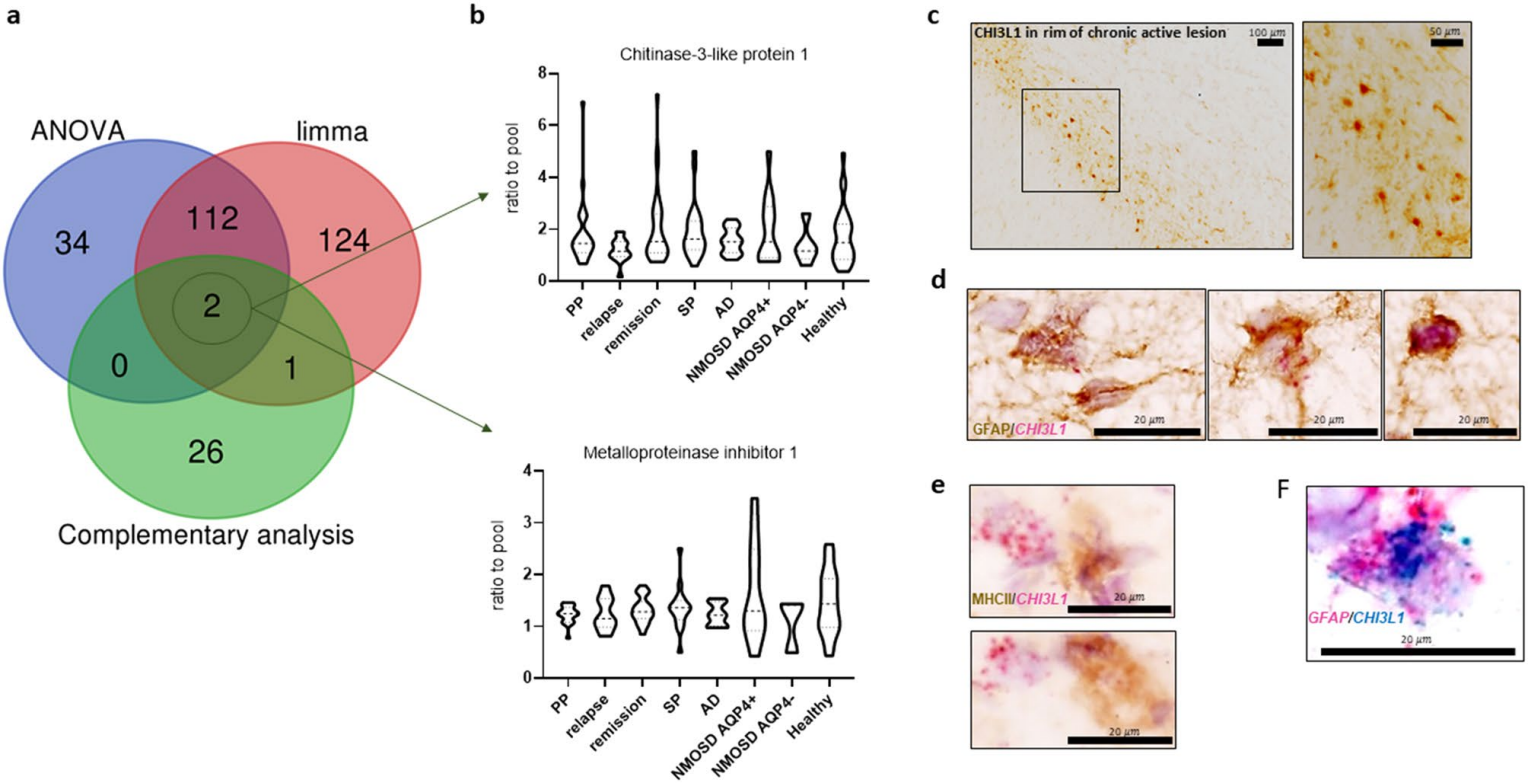
Chitinase-3-like protein 1 (CHI3L1) and metalloproteinase inhibitor 1 (TIMP-1). (**a**) Venn diagram showing the 299 proteins from discovery CSF proteomics significantly altered compared to healthy by different statistical analyses (ANOVA, limma, complementary analysis, see Methods). (**b**) Quantitative levels of chitinase-3-like protein 1 (CHI3L1) and metalloproteinase inhibitor 1 (TIMP-1) in individual CSF samples (detection of CHI3L1: PP = 30 of 30; relapse = 14 of 14; remission = 33 of 33; SP = 26 of 26; AD = 22 of 22; AQP4^+^ NMOSD = 13 of 13; AQP4^−^ NMOSD = 5 of 5; healthy = 27 of 27) (detection of TIMP-1: PP = 19 of 30; relapse = 10 of 14; remission = 27 of 33; SP = 16 of 26; AD = 13 of 22; AQP4^+^ NMOSD = 10 of 13; AQP4^−^ NMOSD = 3 of 5; healthy = 16 of 27). (**c**) Protein expression of CHI3L1 in the rim of a chronic active lesion of progressive MS brain. (**d**) Protein expression of GFAP (brown) and RNA expression of CHI3L1 (red) in the same cells (combined immunohistochemistry and RNAscope). (**e**) Protein expression of MHCII (brown) and RNA expression of CHI3L1 (red) in different cells close to each other at the rim of the lesion (combined immunohistochemistry and RNAscope). (**f**) Co-localized RNA expression of GFAP (red) with CHI3L1 (green) by RNAscope. *PP/SP* primary/secondary progressive multiple sclerosis, *AD* Alzheimer disease, *NMOSD AQP4*^*+/−*^ neuromyelitis optica spectrum disorders serum positive/negative for aquaporin-4 antibody, *AD* Alzheimer disease.

By combining three different statistical analyses (ANOVA, limma, complementary analysis) of the pooled CSF samples, we selected 299 dysregulated proteins (Fig. 4a, Supplementary Table S2). These were quantified in 170 individual CSF samples by mass spectrometry. Two proteins, chitinase-3-like protein 1 (CHI3L1) and metalloproteinase inhibitor 1 (TIMP1) were significantly altered in the pooled samples by all three statistical tests, although they were not significantly altered in the individual samples by the quantitative proteomics (Fig. 4b). However, some of the PPMS and RRMS/remission patients had increased levels of CHI3L1, while some of the AQP4-IgG^+^ NMOSD patients had increased levels of TIMP1 (Fig. 4b). By immunohistochemistry, we also found CHI3L1 expressed at the rim of chronic active lesions in the MS brain (Fig. 4c). The morphology of cells expressing CHI3L1 in chronic active lesions was consistent with astrocytes. The astrocytic expression was confirmed by combined RNAscope and immunohistochemistry that co-localized CHI3L1 and GFAP at the chronic active rim in close proximity to MHCII expressing cells (Fig. 4d–f).

**Figure 5:**
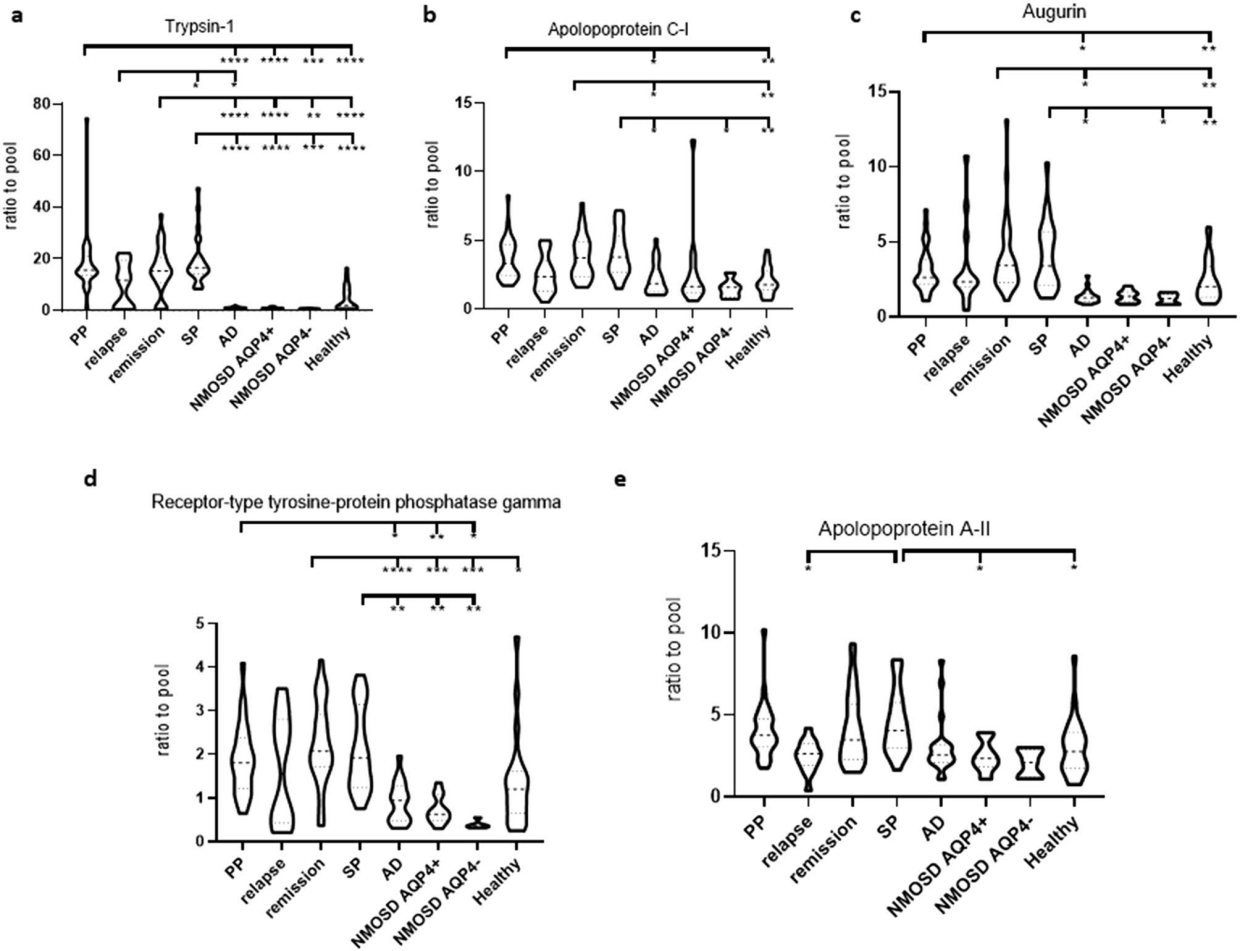
Significantly upregulated unique molecular markers in the CSF of MS subtypes. Overview of 5 proteins significantly upregulated in the CSF of MS subtypes compared to other CNS diseases and healthy controls. (**a**) Trypsin-1 that was significantly upregulated in remission, PPMS and SPMS compared to disease- and healthy controls in individual samples (detection: PP = 30 of 30; relapse = 14 of 14; remission = 33 of 33; SP = 26 of 26; AD = 22 of 22; NMOSD AQP4^+^ = 13 of 13; NMOSD AQP4^−^ = 5 of 5; healthy = 27 of 27). (**b**) Apolipoprotein C-I and (**c**) augurin were significantly upregulated in remission, PPMS and SPMS compared to AD and healthy controls in individual samples. (detection: PP = 30 of 30; relapse = 14 of 14; remission = 33 of 33; SP = 26 of 26; AD = 22 of 22; NMOSD AQP4^+^ = 13 of 13; NMOSD AQP4^−^ = 5 of 5; healthy = 27 of 27). (**d**) Receptor-type tyrosine-protein phosphatase gamma was significantly upregulated in remission, PPMS and SPMS compared to disease controls (detection: PP = 25 of 27; relapse = 12 of 14; remission = 27 of 33; SP = 20 of 26; AD = 18 of 22; NMOSD AQP4^+^ = 11 of 13; NMOSD AQP4^−^ = 5 of 5; healthy = 22 of 27). (**e**) Apolipoprotein A-II was uniquely significantly upregulated in SPMS compared to MS in relapse, NMOSD AQP4^+^ and healthy controls (detection: PP = 30 of 30; relapse = 14 of 14; remission = 33 of 33; SP = 26 of 26; AD = 22 of 22; NMOSD AQP4^+^ = 13 of 13; NMOSD AQP4^−^ = 5 of 5; healthy = 27 of 27). *PP/SP* primary/secondary progressive multiple sclerosis, *AD* Alzheimer disease, *NMOSD AQP4*^*+/−*^ neuromyelitis optica spectrum disorder positive/negative for aquaporin-4 antibody, *AD* Alzheimer disease.

Trypsin-1 protein was the most significantly upregulated protein in RRMS/remission, PPMS and SPMS compared to both the disease- and healthy controls (Fig. 5a). Apolipoprotein C-I and augurin were also upregulated in these three MS subtypes compared to healthy controls and AD patients (Fig. 5b,c). Receptor-type tyrosine-protein phosphatase gamma was also upregulated in these three MS subtypes compared to disease controls (Fig. 5d). Apolipoprotein A-II was significantly upregulated in SPMS compared to relapsing MS, AQP4-IgG^+^ NMOSD and healthy controls (Fig. 5e).

**Figure 6:**
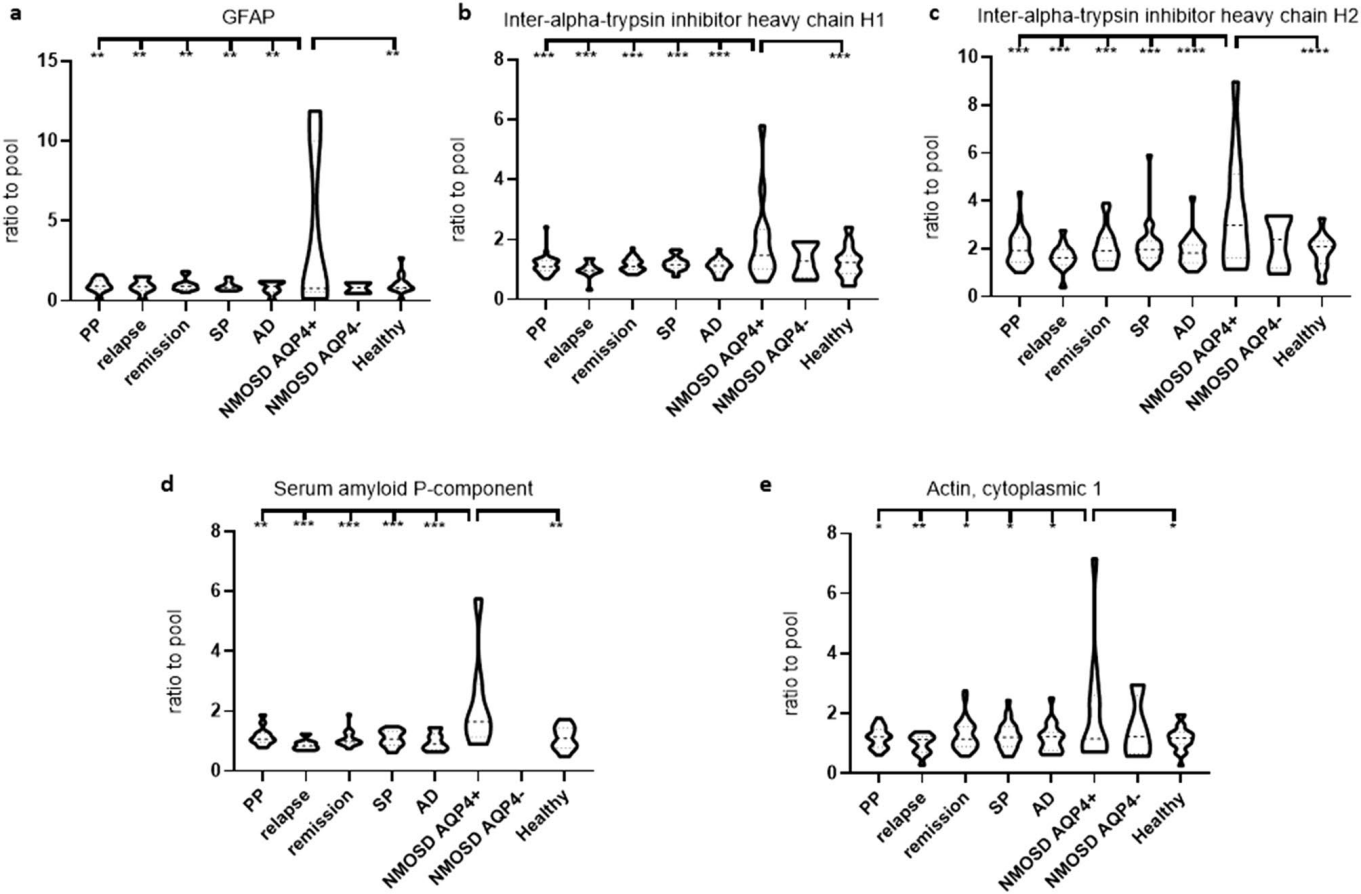
Significantly upregulated unique molecular markers in the CSF of AQP4-IgG + NMOSD. Five proteins were uniquely upregulated in the CSF of patients with AQP4-IgG^+^ NMOSD compared to OND and healthy controls. (**a**) Glial fibrillary acidic protein (GFAP) (detection: PP = 12 of 30; relapse = 8 of 14; remission = 13 of 33; SP = 10 of 26; AD = 8 of 22; NMOSD AQP4-IgG^+^ = 9 of 13; NMOSD AQP4-IgG^−^ = 2 of 5; healthy = 23 of 27). (**b**) Inter-alpha-trypsin inhibitor heavy chain H1 (detection: PP = 30 of 30; relapse = 14 of 14; remission = 33 of 33; SP = 26 of 26; AD = 22 of 22; NMOSD AQP4-IgG^+^ = 13 of 13; NMOSD AQP4-IgG^−^ = 5 of 5; healthy = 27 of 27). (**c**) Inter-alpha-trypsin inhibitor heavy chain H2 (detection: PP = 30 of 30; relapse = 14 of 14; remission = 33 of 33; SP = 26 of 26; AD = 22 of 22; NMOSD AQP4-IgG^+^ = 13 of 13; NMOSD AQP4-IgG^−^ = 5 of 5; healthy = 27 of 27). (**d**) Serum amyloid P-component (detection: PP = 13 of 30; relapse = 7 of 14; remission = 16 of 33; SP = 14 of 26; AD = 10 of 22; NMOSD AQP4-IgG^+^ = 7 of 13; NMOSD AQP4-IgG^−^ = 1 of 5; healthy = 12 of 27). (**e**) Actin, cytoplasmic 1 (detection: PP = 26 of 30; relapse = 13 of 14; remission = 30 of 33; SP = 23 of 26; AD = 19 of 22; NMOSD AQP4-IgG^+^ = 12 of 13; NMOSD AQP4-IgG^−^ = 4 of 5; healthy = 23 of 27). *CSF* cerebrospinal fluid, *NMOSD AQP4-IgG*^*+/−*^ neuromyelitis optica spectrum disorder positive for aquaporin-4 antibody, *AD* Alzheimer disease.

GFAP, inter-alpha-trypsin inhibitor heavy chain H1, and H2, serum amyloid P-component, and actin cytoplasmic 1 protein were uniquely upregulated in AQP4-IgG^+^ NMOSD compared to all MS subtypes, AD and healthy controls (Fig. 6). Glial fibrillary acidic protein/GFAP was detected only in less than 50% of the patients with AQP4-IgG^-^ NMOSD similarly to MS and AD.

### CSF proteome signatures in MS brain lesion transcriptomes

**Figure 7:**
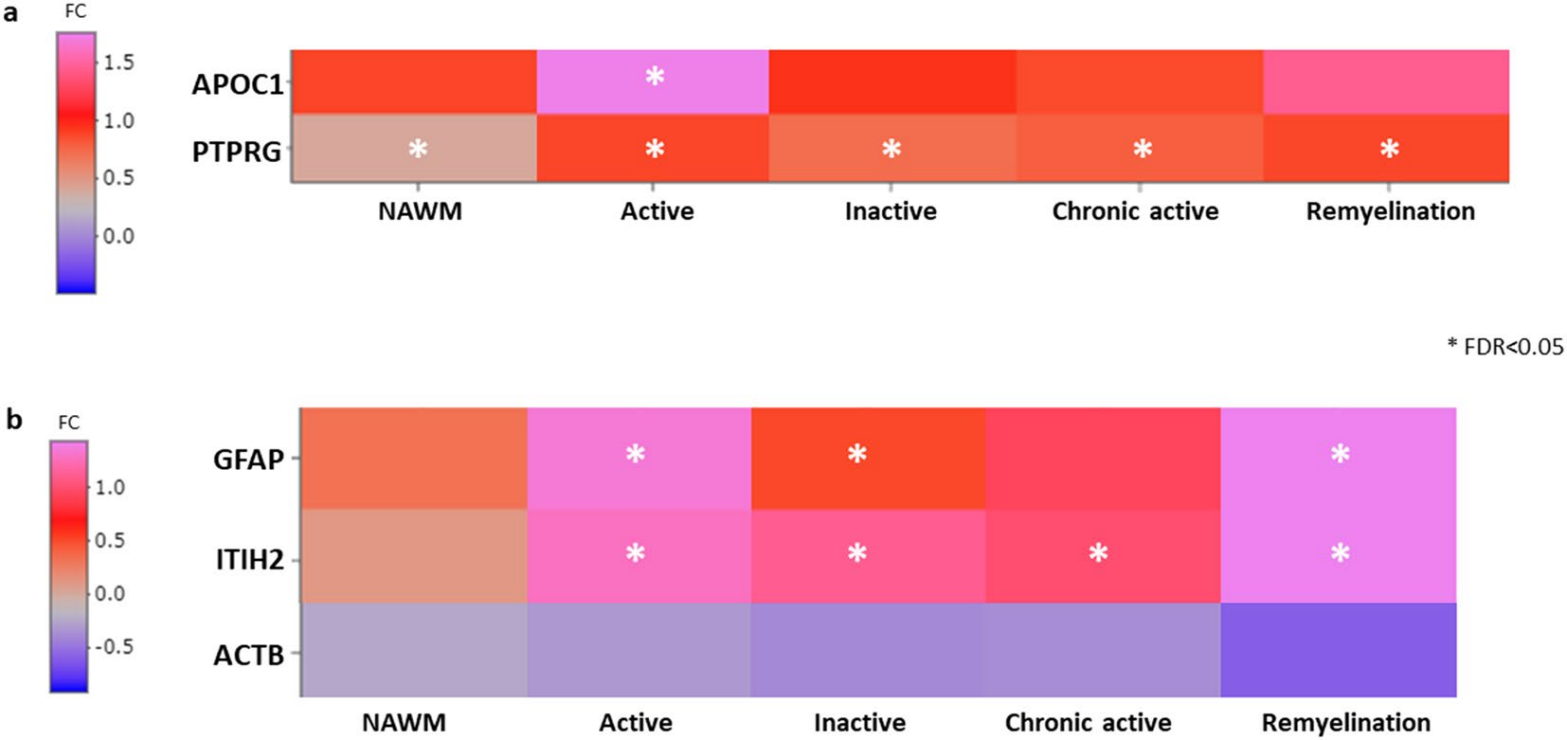
Expression of genes of upregulated disease-specific CSF proteins in transcriptomes of different MS brain lesions. The heatmaps show genes encoding the significantly altered CSF proteins that could be detected in different the brain lesions and normal-appearing white matter (NAWM) of MS. (**a**) The transcripts *APOC1* and *PTPRG* encoding two MS-specific CSF proteins: apolipoprotein C-I and receptor-type tyrosine-protein phosphatase gamma. (**b**) The transcripts *GFAP*, *ITH2* and *ACTB* encoding the NMOSD-specific CSF proteins: glial fibrillary acidic protein (GFAP), inter-alpha-trypsin inhibitor heavy chain H2, and actin cytoplasmic 1. Stars represent significantly differentially expressed genes (FDR < 0.05) in the MS lesions compared to non-neurological disease brains. Colour represents the log2fold change (FC).

We compared the CSF proteome signatures to the recently established transcriptome signatures of different MS lesion types (www.msatlas.dk)^17^. Two of the MS-specific upregulated proteins were present as transcripts in the human MS brain: apolipoprotein C-I (*APOC1*) was significantly upregulated in active lesions, and receptor-type tyrosine-protein phosphatase gamma (*PTPRG*) was significantly upregulated in all WM tissue (NAWM and lesions) (Fig. 7a).

Three of the five altered proteins in AQP4-IgG^+^ NMOSD patients were also detected as transcripts in the MS brain tissue: glial fibrillary acidic protein (*GFAP*) was upregulated in active, inactive and remyelinating lesion types, inter-alpha-trypsin inhibitor heavy chain H2 (*ITIH2*) was significantly upregulated in all lesion types, while actin cytoplasmic 1 (*ATCB*) was not differently expressed compared to non-neurological-disease WM brain areas (Fig. 7b).

## Discussion

This comprehensive two-stage proteomic study with a high number of human CSF samples (n = 170) from a spectrum of different neurological diseases provided information about the global CSF proteomic landscape in MS subtypes compared to inflammatory/degenerative CNS disease controls and healthy controls.

With F-test based feature selection, a combination of 11 proteins could separate CNS diseases without overlap and technical batch effect. Almost half of them were involved in axon-related processes (RTN4R, CNTNAP4, ADAM22, PCSK1N, NRXN1), which could indicate that the neurodegenerative mechanisms may be different between these brain diseases. All the transcripts coding for the 11 proteins could also be detected in the brain^10^ (msatlas.dk), indicating that they originated from the brain tissue and not from the systemic peripheral compartment. Chronic active lesion type had the highest number of significantly increased transcripts coding for the 11 proteins. This lesion type dominates and is increased in SPMS, thereby the combination of the brain transcriptome and CSF proteome suggests a uniqueness of SPMS. Among the 11 proteins, the contactin-associated protein-like 4 is involved in the formation and maintenance of myelinated axons^18^, and its transcript (*CNTNAP4*) was upregulated in the inactive lesion type; olfactomedin-like protein 3, a known marker of activated ramified microglia, and *OLFML3* was also significantly upregulated in chronic active lesions and in NAWM; neurorexin-1 can be related to neurodegeneration in MS^19^, and *NRXN1* was uniquely significantly upregulated in the chronic active lesion type associated with progressive MS.

The combination of 8 additional proteins could also separate the MS subgroups, and 4 were related to intracellular processing and transporting of synthesized proteins and lipids (*GOLM1, NUCB1, NPC2, SELENBP1*). We detected transcripts coding for the 8 proteins in the brain, except hemoglobin subunit alfa (Fig. 2d,f). This may again suggests that 7 of these 8 proteins distinguishing the MS subtypes may not come from the systemic compartment, but instead reflect different events of the brain pathology. One of them, selenium-binding protein 1 is an astrocytic marker related to metabolic processes^20^, and *SELENBP1* was uniquely expressed in inactive lesions characterized by astrocytic scar tissue^10^. Another was the secreted frizzled-related protein 3, involved in axon targeting basement membrane breakdown^21^, and the *FRZB* gene was significantly upregulated in active and chronic active lesion types. This molecular CSF profile and associated brain lesion spectrum highlights the importance of non-inflammatory mechanisms in differentiating both CNS diseases and MS subtypes.

Overall, the CSF proteome seemed to be most unique for SPMS and AQP4-IgG^+/−^NMOSD based on both the separability between pre-defined groups (Fig. 2) and the differential abundance of proteins between groups (Fig. 3a). These two diseases also had the highest number of significantly altered proteins compared to the proteome of healthy controls (FDR < 0.001) (Fig. 3b).

We also examined pathways that were different among diseases and MS subtypes (Fig. 3c,d). In this regard, RRMS/relapse was the most distinct disease group with almost nothing in common with the other diseases. It was dominated by unique immune-related pathways, and the top predicted diseases/functions were more related to systemic than CNS-specific events. The unique SPMS enriched pathway was the “Coagulation” system, while PPMS and AD shared “Intrinsic Prothrombin Activation Pathway”. A previous study also found proteins involved in coagulation unique to chronic active lesion samples, suggesting dysregulation of molecules associated with coagulation in chronic active lesions^22^. Another recent study also identified higher levels of CSF proteins related to the coagulation cascade in MS patients with higher cortical lesion load^9^.

Unexpectedly, in our study immune related proteins such as cytokines, chemokines, growth factors and adhesion molecules were not frequently detected. This could be because of the constrained dynamic range of mass spectrometers to truly cover the broad spectrum of lower abundance or because the cytokine and chemokine amount is not the true strong dominator when examining the global proteome differences between neuroinflammatory diseases. A systematic review revealed 19 inflammatory proteins specifically altered in MS^23^. Not surprisingly, the majority of the upregulated MS proteins (11 of 19) were immunoglobulins. Another recent review reported several potential markers^23^. In line with the review, we also detected five potential NMOSD markers significantly increased in our NMOSD samples including GFAP, haptoglobin, C5, factor H, and C1inh. Additionally, sVCAM-1 was significantly increased in the CSF of PPMS patients by the non-targeted proteomics.

Next, to search for individual disease-specific molecular markers, 299 proteins were selected and quantified in 170 individual CSF samples (majority of these were also used for the discovery phase). Two proteins (CHI3L1 and TIMP1) were significantly altered in all three statistical tests (ANOVA, limma, complementary analysis) in the pooled discovery CSF proteome, but were not unique to diseases in the individual quantification study. However, a subgroup of MS patients with PP and remission had increased levels of CHI3L1 (Fig. 4b). CHI3L1 (YKL-40) is a promising biomarker of inflammation in progressive MS^24^, and was originally discovered in the CSF proteome of patients with CIS converting to RRMS^16^. Immunohistochemistry and RNAscope indicated that the gene encoding CHI3L1 was primary expressed by astrocytes in the rim of chronic active lesions (Fig. 4c–f). Another recent study also found that CHI3L1 reflects disease progression, and together with the biomarker neurofilament light chain protein, it may help to discriminate MS phenotypes^25^. These data suggest that some of the emerging biomarkers in progressive MS may reflect unique molecular changes in the brain related to specific subtypes of lesions and thereby a possible distinct pathogenesis. The high expression of CHI3L1 in the CSF of patients with progressive MS^26^ may be related to the increasing number of a specific subtype of chronic active lesions, and we may speculate that its level in the CSF of patients with progressive MS may even reflect the number of this lesion type in the brain. The expression of CHI3L1 by astrocytes has been recently described in neurodegenerative diseases and often appears in clusters of astrocytes^27^. Knock-out animal models indicated a protective role of CHI3L1, as traumatic brain injury and experimental autoimmune encephalomyelitis were more severe in its absence^28,29^. CH13L1 can also influence the migratory capacity of astrocytes and reduces astrogliosis^28,29^. It may therefore dampen the inflammation and limit astrogliosis.

TIMP-1 seemed to be highly expressed in a subset of AQP4-IgG^+^ NMOSD patients (Fig. 4b). TIMP-1 is produced by astrocytes in both homeostasis and early/acute inflammatory events^30^. We have previously found that TIMP-1 peaked during acute remyelination in the cuprizone model and was associated with reduced inflammation in the CSF of MS^8^. Induction of TIMP-1 in neurons and astrocytes was also related to early cellular events triggered by seizures and with long-lasting changes in tissue reorganization and/or neuroprotection^31^. Increased TIMP-1 levels in serum has also been proposed as a prognostic biomarker of mortality in brain trauma injury patients^32^. The presence of TIMP-1 in brain of AQP4-IgG^+^ NMOSD patients would be interesting to investigate, but postmortem brain tissue of those patients are lacking. In all, increased TIMP-1 and CH13L1 in the CSF may reflect acute and chronic astrocytic responses in subgroups of MS and AQP4-IgG^+^ NMOSD patients.

The targeted proteomics of 299 proteins identified 10 upregulated molecular markers specific to MS and AQP4-IgG^+^ NMOSD.

Two apolipoproteins were increased in the CSF proteome of MS. These are important players in cholesterol homeostasis, and in CNS diseases for neuronal homeostasis and regeneration^33^. Apolipoprotein C-I was significantly upregulated in RRMS in remission, PPMS and SPMS, and its transcript was significantly induced in active MS lesions in SPMS brain (Fig. 7a). Apoprotein A-II was significantly altered in the CSF in SPMS compared to both AQP4-IgG^+^ NMOSD and healthy controls (Fig. 6e). Increased levels of apoprotein A-II has been associated with fatigue in MS patients^34^, and it may reflect later disease mechanisms accumulated with chronic damage. Apolipoproteins have also been linked to the genetic risk of MS: APOE genotype has been associated with disease severity and MR activity^35–37^.

Trypsin-1, a protease that degrade other proteins, was also significantly upregulated in remission, PP and SPMS compared to the disease- and healthy controls. We were not able to detect the gene of this protein (*PRSS1*) expressed in the MS brain^10^, but it is normally produced in pancreas and activated in duodenum and intestinal lumen, where it further activate enzymes for digestion. However, the protein activating trypsin from trypsinogen (endopeptidase) has also been found expressed in the brain^38^ and trypsin activates proteins also suggested as MS biomarkers as kallikreins^39–41^. However, the potential presence and function of trypsin-1 in the CNS are unclear.

Receptor-type tyrosine-protein phosphatase gamma (*PTPRG*) levels were increased RRMS in remission, PP and SPMS compared to the disease controls (Fig. 6d). Another study also found increased levels in the CSF of early MS patients compared to controls^42^, suggesting that it may be induced from disease onset. We also found that its transcript was significantly upregulated in progressive MS tissue in both NAWM and all kind of lesions (Fig. 7a).

Lastly, we noticed that 5 proteins were upregulated uniquely in the CSF of patients with AQP4-IgG^+^ NMOSD. Increased GFAP reflects astrocyte damage and death in AQP4-IgG^+^ NMOSD^43,44^. It was not increased in AQP4-IgG seronegative NMOSD indicating that at least in a subset of these patients the disease mechanisms do not primarily target astrocytes. Another study also reported higher GFAP levels in AQP4-IgG^+^ patients compared to AQP4-IgG^−^ NMOSD^45^. The other 4 upregulated unique proteins in the CSF may not be related to astrocytes, as their transcripts were not enriched in astrocyte signatures^46^. The unique elevation of serum amyloid P-component in the CSF in AQP4-IgG^+^ NMOSD may be related to passive transfer because of damage of the blood–brain barrier^47^. Upregulation of inter-alpha-trypsin inhibitor heavy chain H1 and H2 may represent endogenous neuroprotective immunomodulatory proteins within the CNS^48^. The *ITIH2* gene was significantly upregulated in all lesion types in the MS brain (Fig. 7b), suggesting that this molecule can be an indicator of non-specific neurological inflammatory damage and control.

A major limitation when examine the disease markers from a range of different CNS diseases, is that it is difficult to distinguish whether the differences are due to lifestyle, the nature of age or sex, or if it reflects differences in disease mechanisms. However, the proteome landscape of AD and relapsing MS were more similar than to other disease groups despite their huge age gap (33.6 ± 10 age and 72.2 ± 7.9 years), indicating that the effect of age might be minor at least in these cohorts. Including age-specific control groups may help to more specifically identify disease-specific changes, or at least reveal, what is the influence of age and sex on disease related markers. The absence of such age-specific controls is a limitation of our study.

In conclusion, with the combination of untargeted and targeted quantitative proteomic analysis of the CSF, we identified molecular markers that differentiated between neuroinflammatory and neurodegenerative CNS diseases, and also MS subtypes. Moreover, the general linear representation of CNS diseases from inflammation to degeneration is more complex, as the proteome of SPMS was the most different from the other subtypes of MS including PPMS, and AD had more in common with NMOSD and RRMS than expected. We could also compare different kind of omics in different kind of compartments as chronic active lesion type, the most distinct lesion type in progressive MS also had highest expression levels of the 11 proteins that made SPMS most unique from all the MS subtypes and the array of controls. With comprehensive bioinformatics 8 proteins not reported before could separate the MS subtypes with their transcripts present in MS lesions. These data may suggest that non-inflammatory pathways in the brain may play an important part to differentiate pathological mechanisms among CNS diseases and even MS subtypes.

## Supplementary Information

Supplementary Figure S1.

Supplementary Figure S2.

Supplementary Figure S3.

Supplementary Table S1.

Supplementary Table S2.

## Data Availability

The mass spectrometry proteomics data have been deposited to the ProteomeXchange Consortium via the PRIDE^49^ partner repository with the dataset identifier PXD017643.
