# Supplementary material for: CSF proteome in multiple sclerosis subtypes related to brain lesion transcriptomes
Source: Sci Rep. 2021 Feb 18;11:4132. doi: 10.1038/s41598-021-83591-5 (PMC7892884; doi:10.1038/s41598-021-83591-5)
Supplement: Supplementary file 1 — Supplementary Figure S1. [file 41598_2021_83591_MOESM1_ESM.pdf]

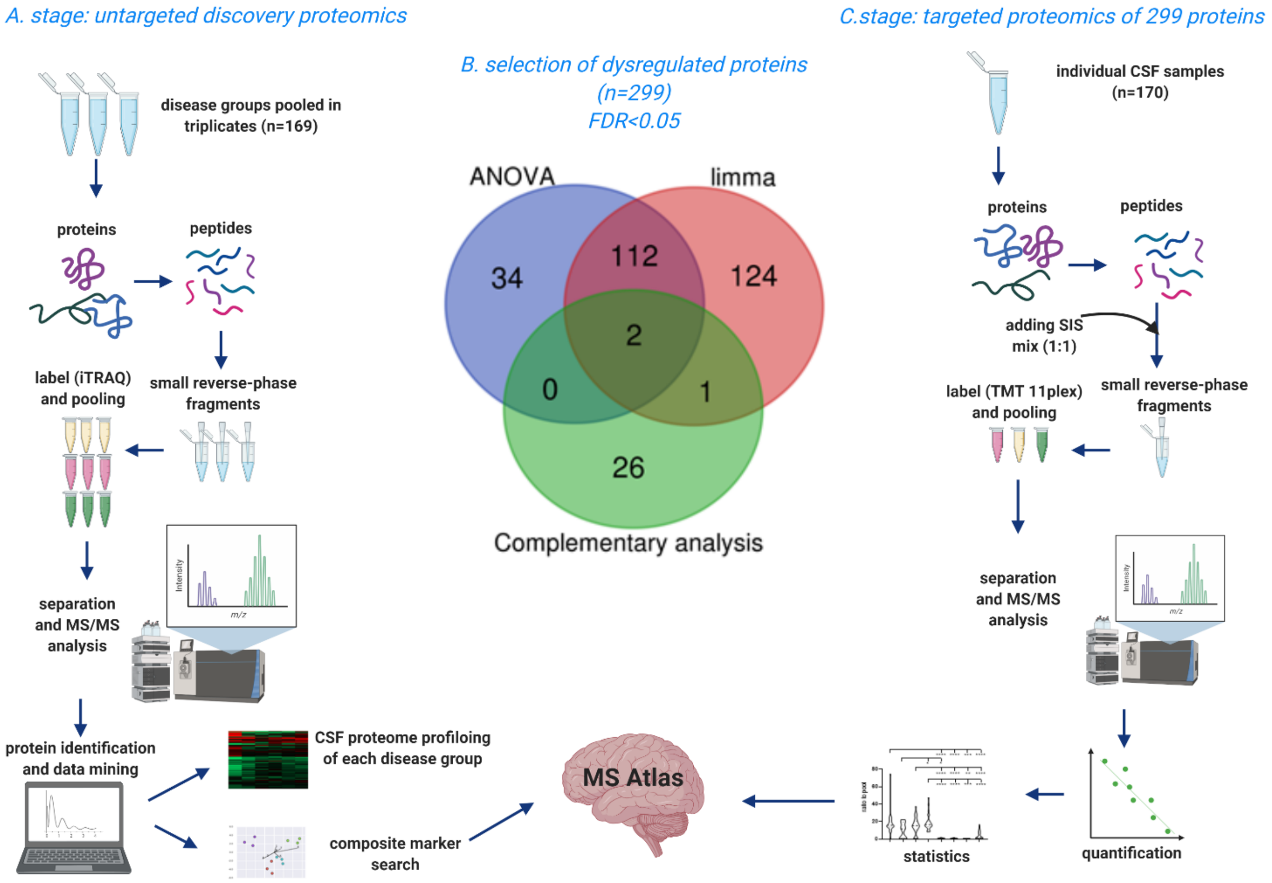

Supplementary Fig. S1: Experimental overview

(A) 169 CSF samples were collected from healthy controls, MS subtypes, and disease controls with different degree of neurodegeneration and inflammation. CSF samples from each group were pooled into three technical replicates, prepared for peptide solution, labelled with iTRAQ 8plex, and distributed into three sets that were analyzed by LC-MS/MS. Raw data was preprocessed, identified proteins were quantified and statistical analyses were performed. The data was examined and visualized with bioinformatic tools. **(B)** Based on different statistical analyses (ANOVA, limma, complementary analysis- see Methods), 299 proteins were selected for quantification in individual samples. **(C)** 170 CSF samples from PPMS (n=30), relapsing MS (n=14), remission MS (n=33), SPMS (n= 26), AD (n=22), NMOSD AQP4-IgG^{+} (n=13), NMOSD AQP4-IgG^{-} (n=5) and healthy (n=27) were used to quantify the 299 proteins in each individual CSF by mass spectrometry labelled with 11TMT plex spiked with SIS,

and analysed by ANOVA. Proteins were grouped based on their specific regulation in disease groups, and their gene expression was also examined in different brain WM lesion types of progressive MS by using MS-Atlas.

CSF: cerebrospinal fluid; PP/SPMS: primary/secondary progressive multiple sclerosis; AD: Alzheimer disease; NMOSD AQP4-IgG$^{+/-}$: neuromyelitis optica spectrum disorder positive/negative for immunoglobulin G antibody against aquaporin-4; LC-MS/MS: Liquid chromatography tandem mass spectrometry; iTRAQ: Isobaric tag for relative and absolute quantitation; TMT: Tandem Mass Tag; SIS: stable isotope standards; OND: other neurological diseases.

The figure was created with BioRender.com.
