# Supplementary material for: CSF proteome in multiple sclerosis subtypes related to brain lesion transcriptomes
Source: Sci Rep. 2021 Feb 18;11:4132. doi: 10.1038/s41598-021-83591-5 (PMC7892884; doi:10.1038/s41598-021-83591-5)
Supplement: Supplementary file 2 — Supplementary Figure S2. [file 41598_2021_83591_MOESM2_ESM.pdf]

A

Patient group ->	PP	Relapse	Remission	SP	AD	NMOSD +	NMOSD -	Healthy	iTRAQ Set
# of persons/pool ->	9	3	11	9	7	5	5	10	A
# of persons/pool ->	10	3	11	9	7	5		11	B
# of persons/pool ->	10	2	11	8	8	4		11	C

B

Channel 126 - Pool

Channel 127_N	Channel 127_C	Channel 128_N	Channel 128_C	Channel 129_N	Channel 129_C	Channel 130_N	Channel 130_C	Channel 131_N	Channel 131_C
Relapse	NMO Ab-	SP	NMO Ab+	PP	AD	PP	Remission	Healthy	Remission
AD	Relapse	PP	NMO Ab-	NMO Ab+	SP	Healthy	PP	Remission	Healthy
Remission	AD	Relapse	PP	Remission	PP	NMO Ab+	Healthy	SP	Remission
SP	PP	Healthy	Relapse	Remission	NMO Ab-	NMO Ab+	AD	Healthy	PP
PP	SP	SP	AD	Relapse	Remission	Remission	PP	NMO Ab+	Healthy
Remission	Healthy	Remission	SP	AD	Relapse	PP	NMO Ab-	PP	NMO Ab+
Healthy	Remission	Healthy	Remission	SP	AD	Relapse	NMO Ab+	SP	PP
Remission	Healthy	Remission	Healthy	AD	NMO Ab+	SP	Relapse	PP	NMO Ab-
Remission	NMO Ab+	AD	PP	Healthy	Remission	Healthy	PP	Relapse	SP
PP	Remission	NMO Ab+	Healthy	PP	Healthy	AD	SP	Remission	Relapse
Relapse	SP	PP	AD	Remission	PP	Healthy	AD	Remission	SP
NMO Ab+	PP	SP	Remission	Relapse	Healthy	Remission	SP	AD	Healthy
SP	NMO Ab+	Healthy	Remission	NMO Ab+	SP	Relapse	PP	Remission	AD
PP	Remission	SP	Remission	SP	Healthy	AD	Relapse	AD	Remission
AD	Healthy	Remission	SP	AD	SP	PP	Remission	Healthy	PP
PP	AD	PP	AD	SP	Healthy	SP	Remission	PP	Healthy
Healthy	SP	AD	PP	PP	Remission	Remission	Healthy	SP	AD

Figure 2, Supporting information: Plate design of the two mass spectrometry experiments

(A) Distribution of samples between the three iTRAQ 8plex sets.

(B) Patient samples randomly distributed across 17 TMT sets. Colours represent different disease/control groups. The pooled group is a mix of all the samples. PP/SP: primary/secondary progressive multiple sclerosis; AD: Alzheimer disease; NMOSD +/- and NMO Ab+, Ab-$^{-}$: neuromyelitis optica spectrum disorder serum positive/negative for immunoglobulin G antibody against aquaporin-4; iTRAQ: isobaric tag for relative and absolute quantitation; TMT: Tandem Mass Tag.
