# Supplementary material for: CSF proteome in multiple sclerosis subtypes related to brain lesion transcriptomes
Source: Sci Rep. 2021 Feb 18;11:4132. doi: 10.1038/s41598-021-83591-5 (PMC7892884; doi:10.1038/s41598-021-83591-5)
Supplement: Supplementary file 3 — Supplementary Figure S3. [file 41598_2021_83591_MOESM3_ESM.pdf]

Additional file 3

Functional analyses of MS subtypes, NMOSD and Alzheimers Disease

Remission MS

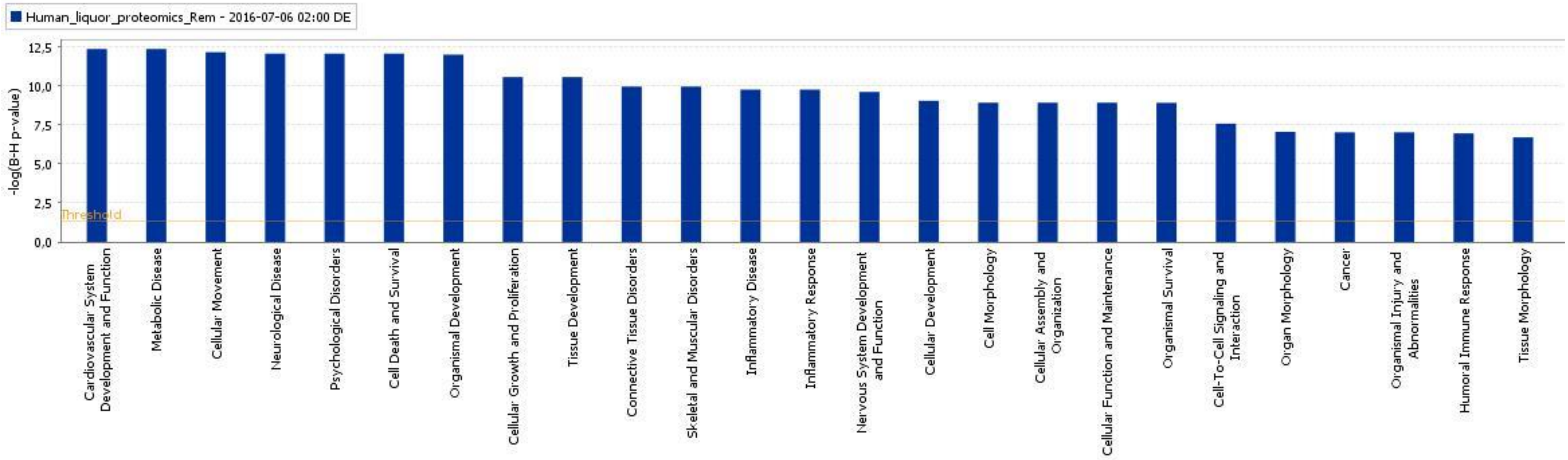
Human_liquor_proteomics_Rem - 2016-07-06 02:00 DE
-log(B-H p-value)
12,5
10,0
7,5
5,0
2,5
0,0
Threshold
Cardiovascular System Development and Function
Metabolic Disease
Cellular Movement
Neurological Disease
Psychological Disorders
Cell Death and Survival
Organismal Development
Cellular Growth and Proliferation
Tissue Development
Connective Tissue Disorders
Skeletal and Muscular Disorders
Inflammatory Disease
Inflammatory Response
Nervous System Development and Function
Cellular Development
Cell Morphology
Cellular Assembly and Organization
Cellular Function and Maintenance
Organismal Survival
Cell-To-Cell Signaling and Interaction
Organ Morphology
Cancer
Organismal Injury and Abnormalities
Humoral Immune Response
Tissue Morphology

Analysis: Human_liquor_proteomics_Rem - 2016-07-06 02:00 DE

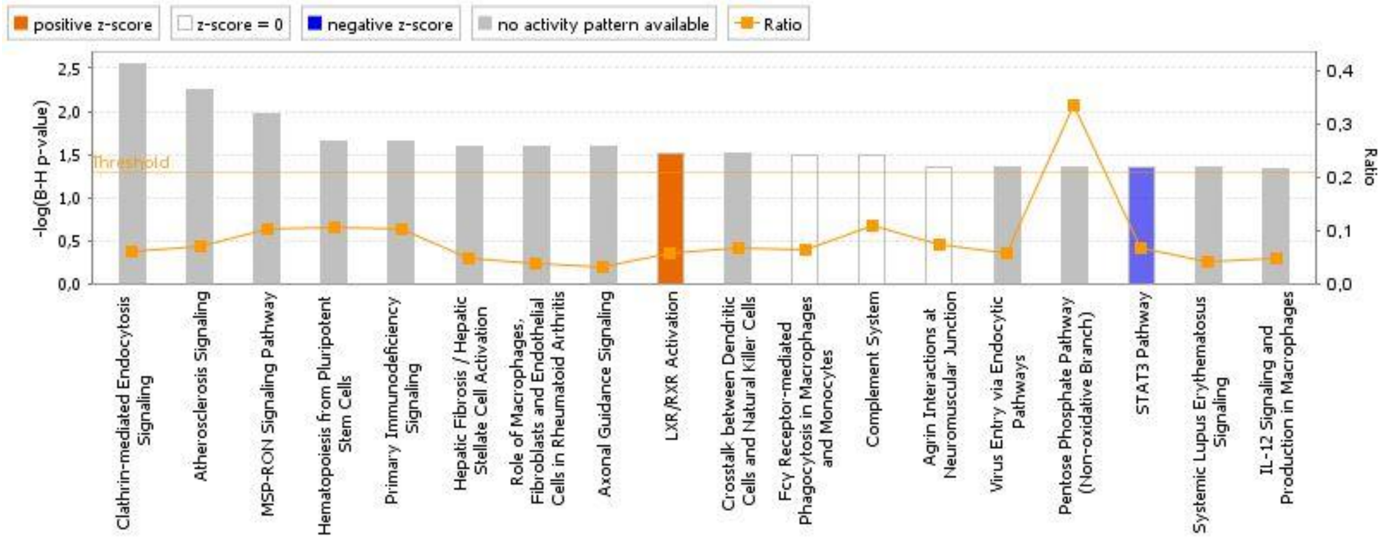

© 2000-2016 QIAGEN. All rights reserved.

Analysis: Human_liquor_proteomics_Rem - 2016-07-06 02:00 DE

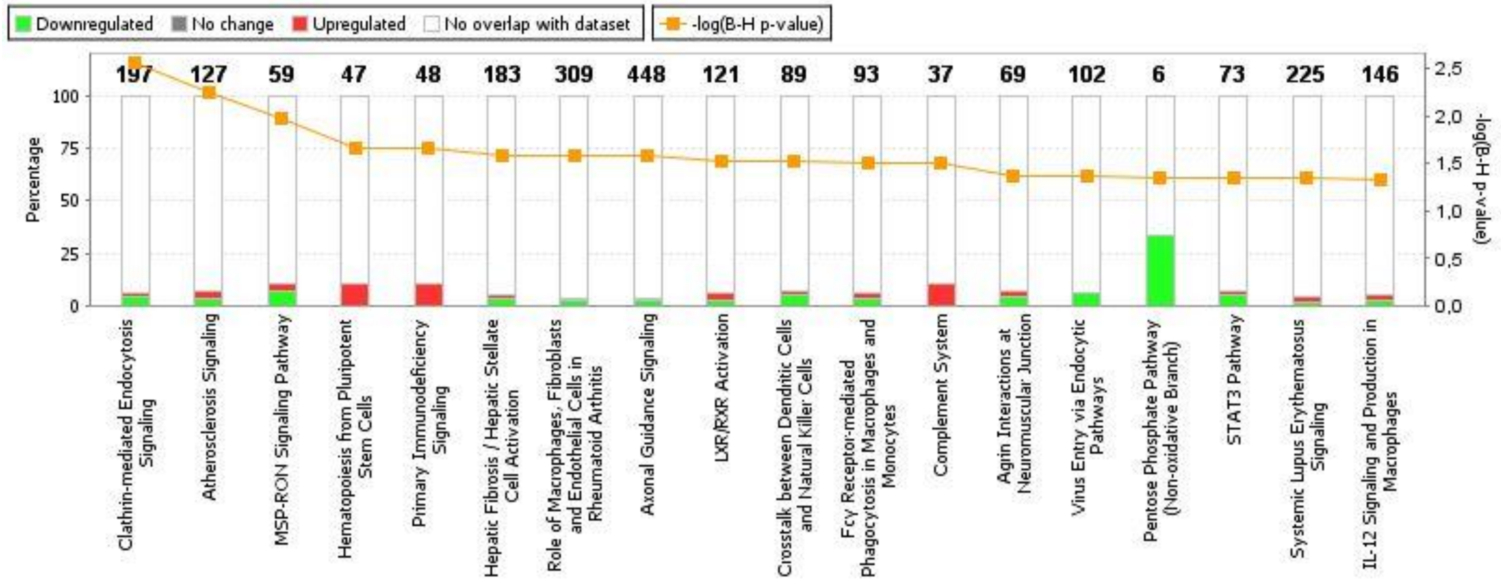

© 2000-2016 QIAGEN. All rights reserved.

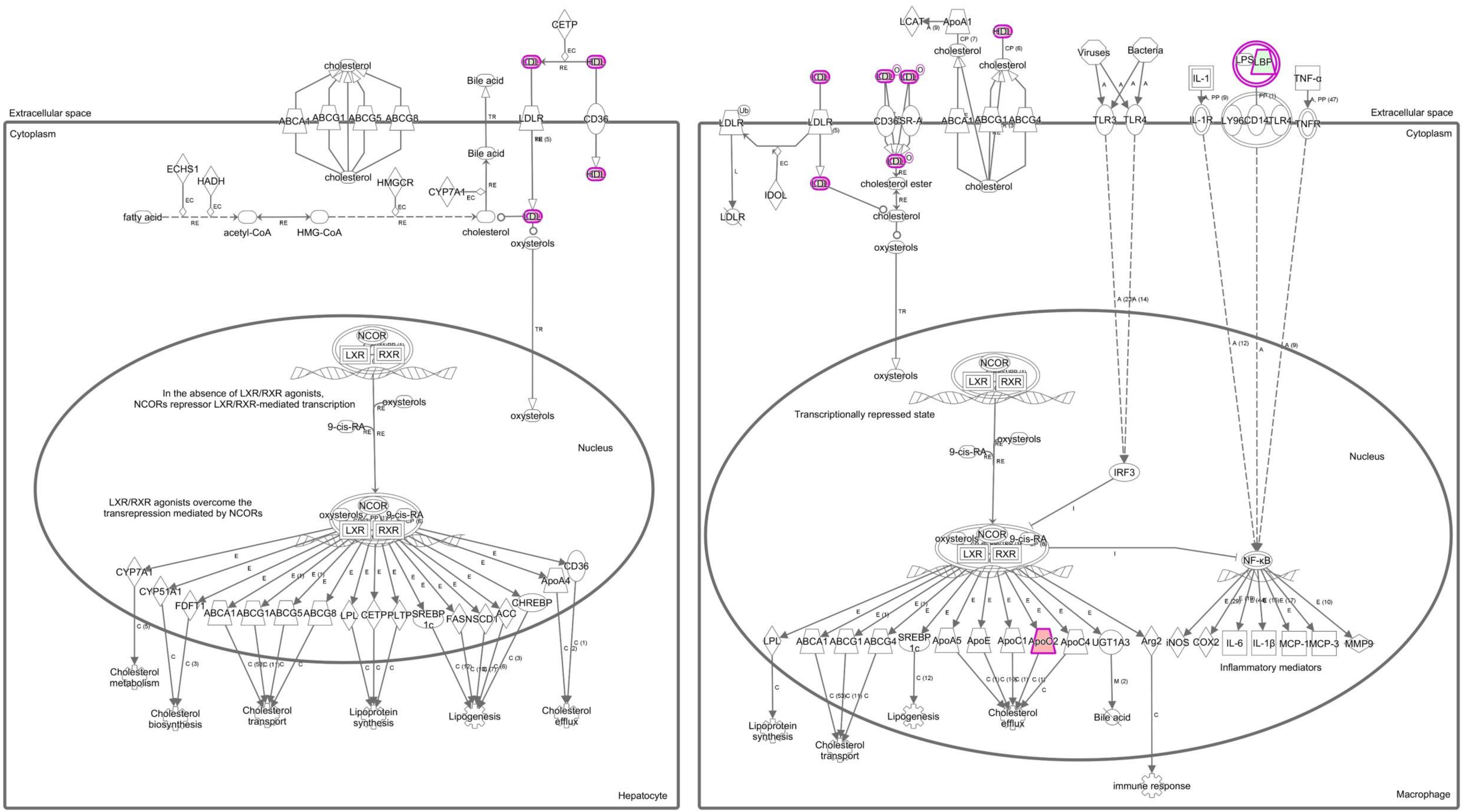

© 2000-2016 QIAGEN. All rights reserved.

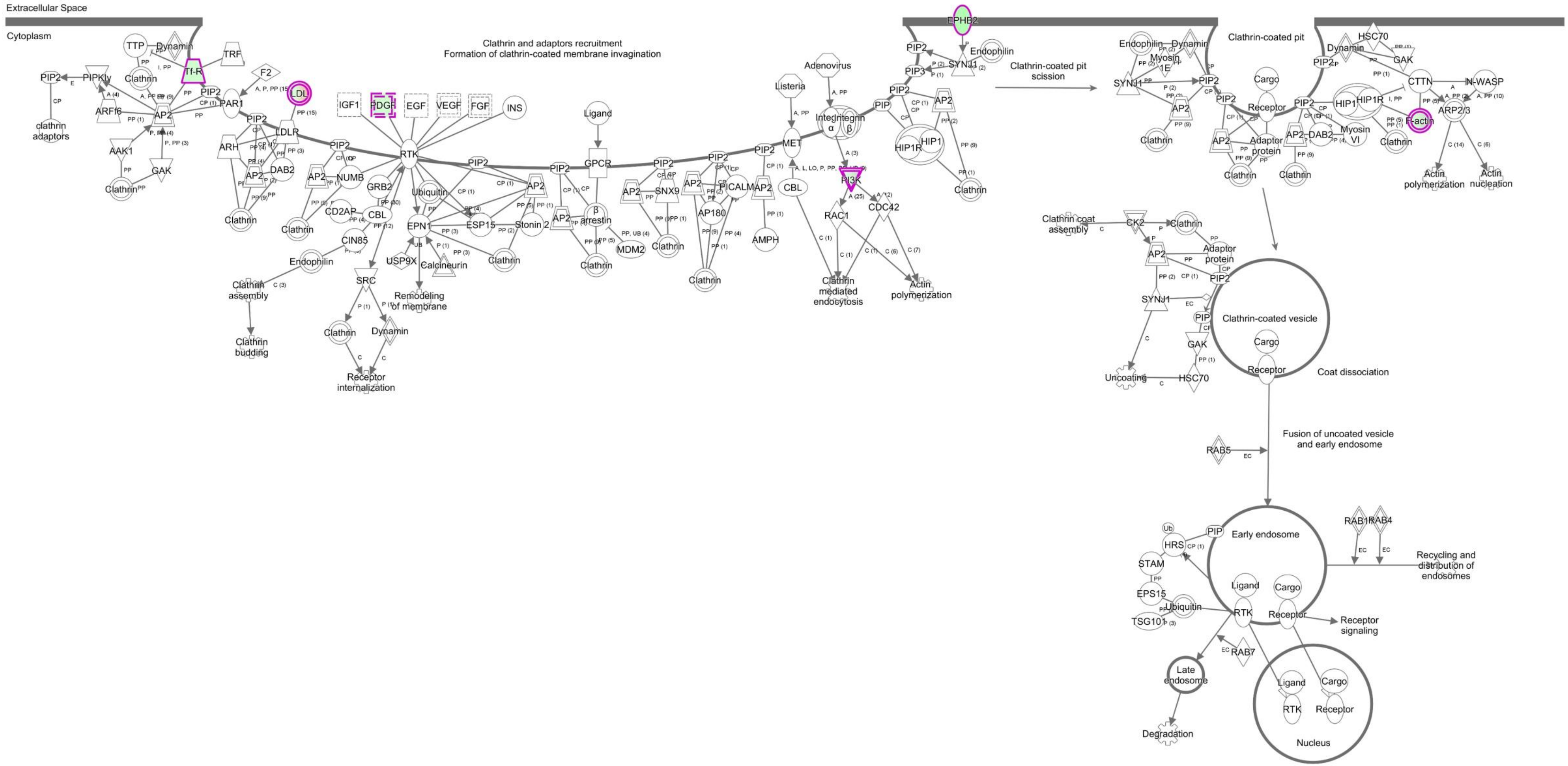

© 2000-2016 QIAGEN. All rights reserved.

Axonal Guidance Signaling

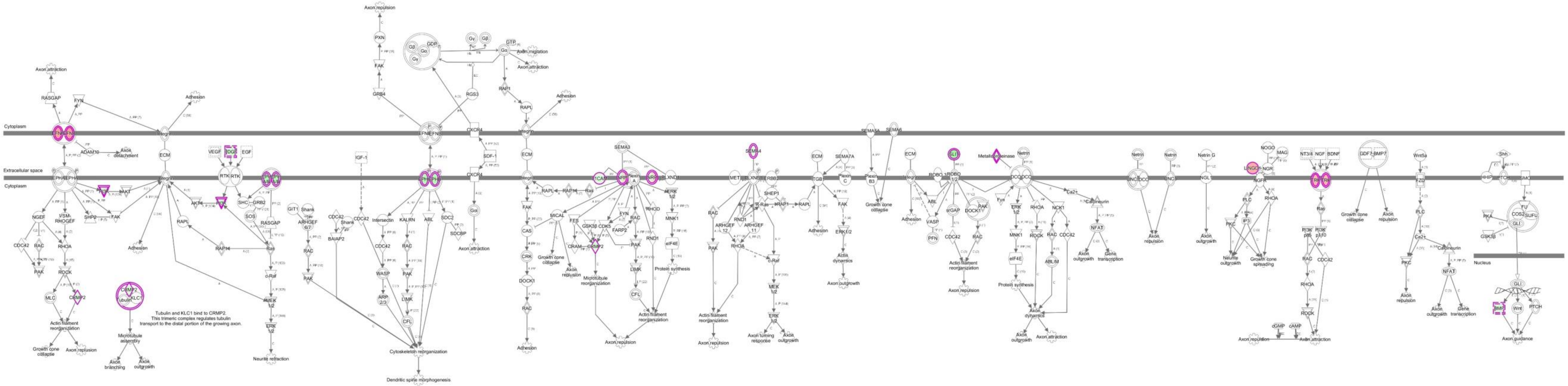

© 2000-2016 QIAGEN. All rights reserved.

Relapse MS

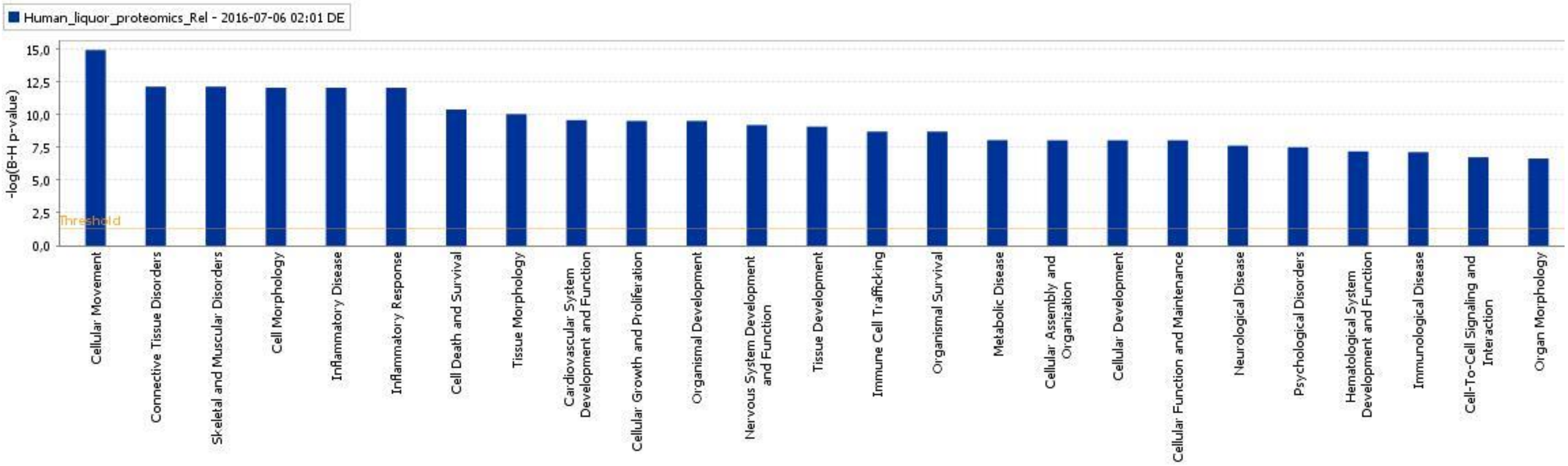

Human_liquor_proteomics_Rel - 2016-07-06 02:01 DE
-log(B-H p-value)
15,0
12,5
10,0
7,5
5,0
2,5
0,0
Threshold
Cellular Movement
Connective Tissue Disorders
Skeletal and Muscular Disorders
Cell Morphology
Inflammatory Disease
Inflammatory Response
Cell Death and Survival
Tissue Morphology
Cardiovascular System Development and Function
Cellular Growth and Proliferation
Organismal Development
Nervous System Development and Function
Tissue Development
Immune Cell Trafficking
Organismal Survival
Metabolic Disease
Cellular Assembly and Organization
Cellular Development
Cellular Function and Maintenance
Neurological Disease
Psychological Disorders
Hematological System Development and Function
Immunological Disease
Cell-To-Cell Signaling and Interaction
Organ Morphology

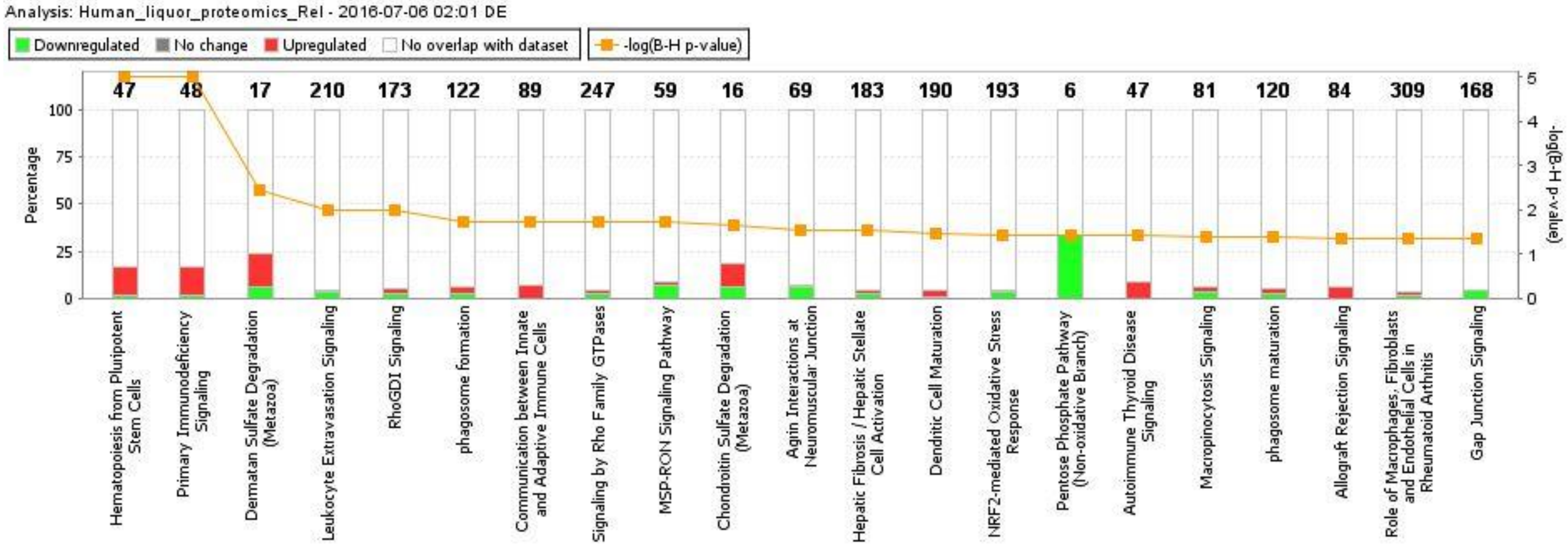

© 2000-2016 QIAGEN. All rights reserved.

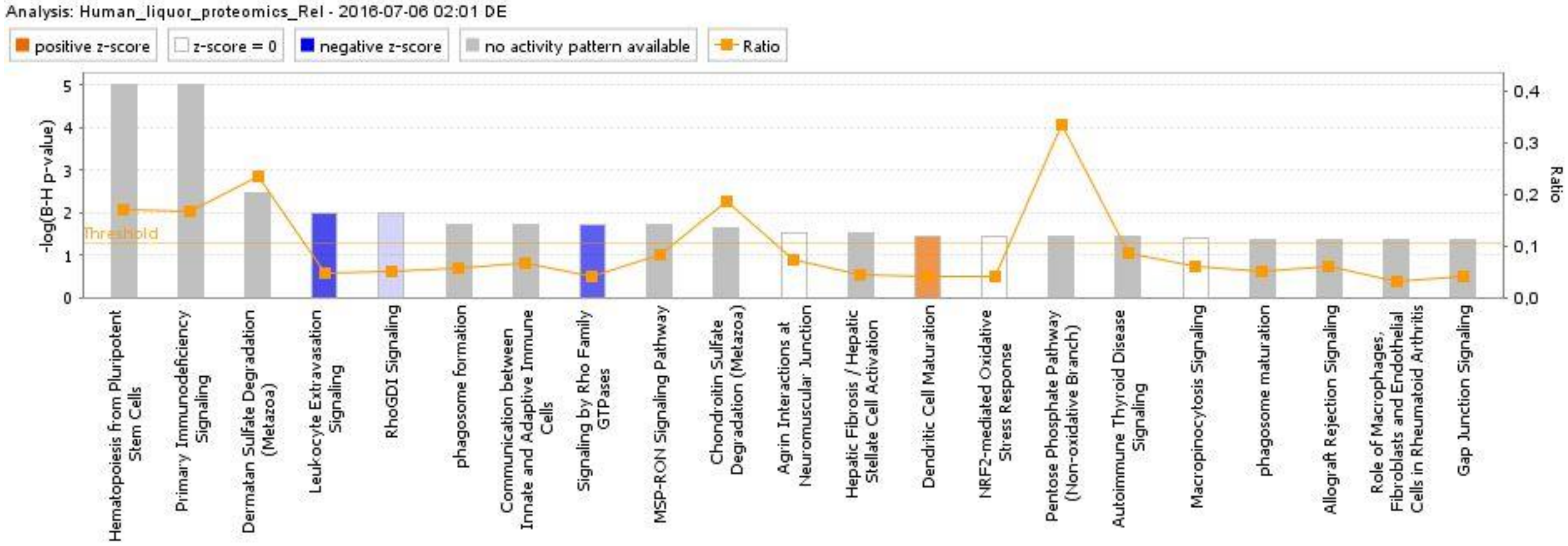

© 2000-2016 QIAGEN. All rights reserved.

Leukocyte Extravasation Signaling

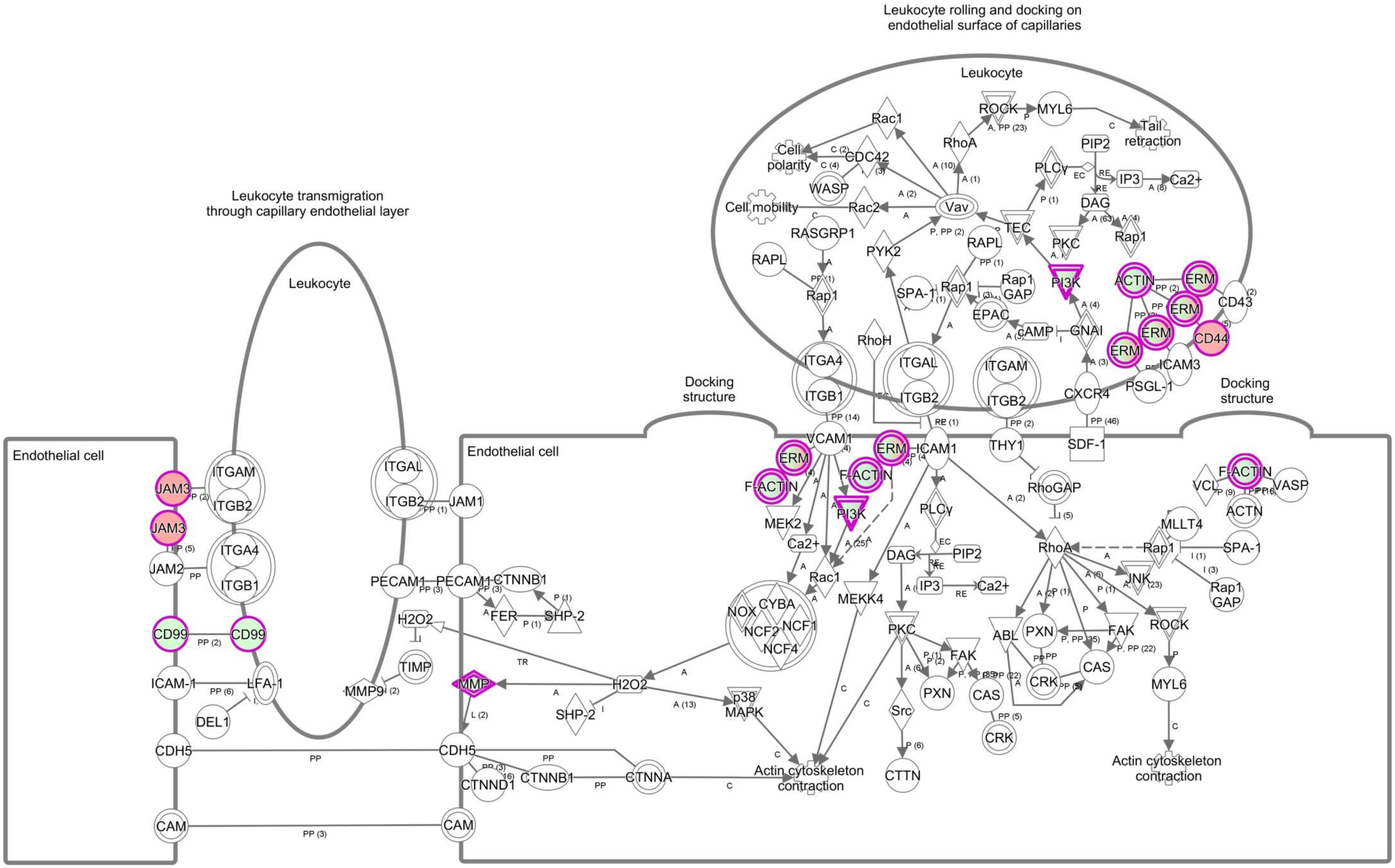

© 2000-2016 QIAGEN. All rights reserved.

Agrin Interactions at Neuromuscular Junction

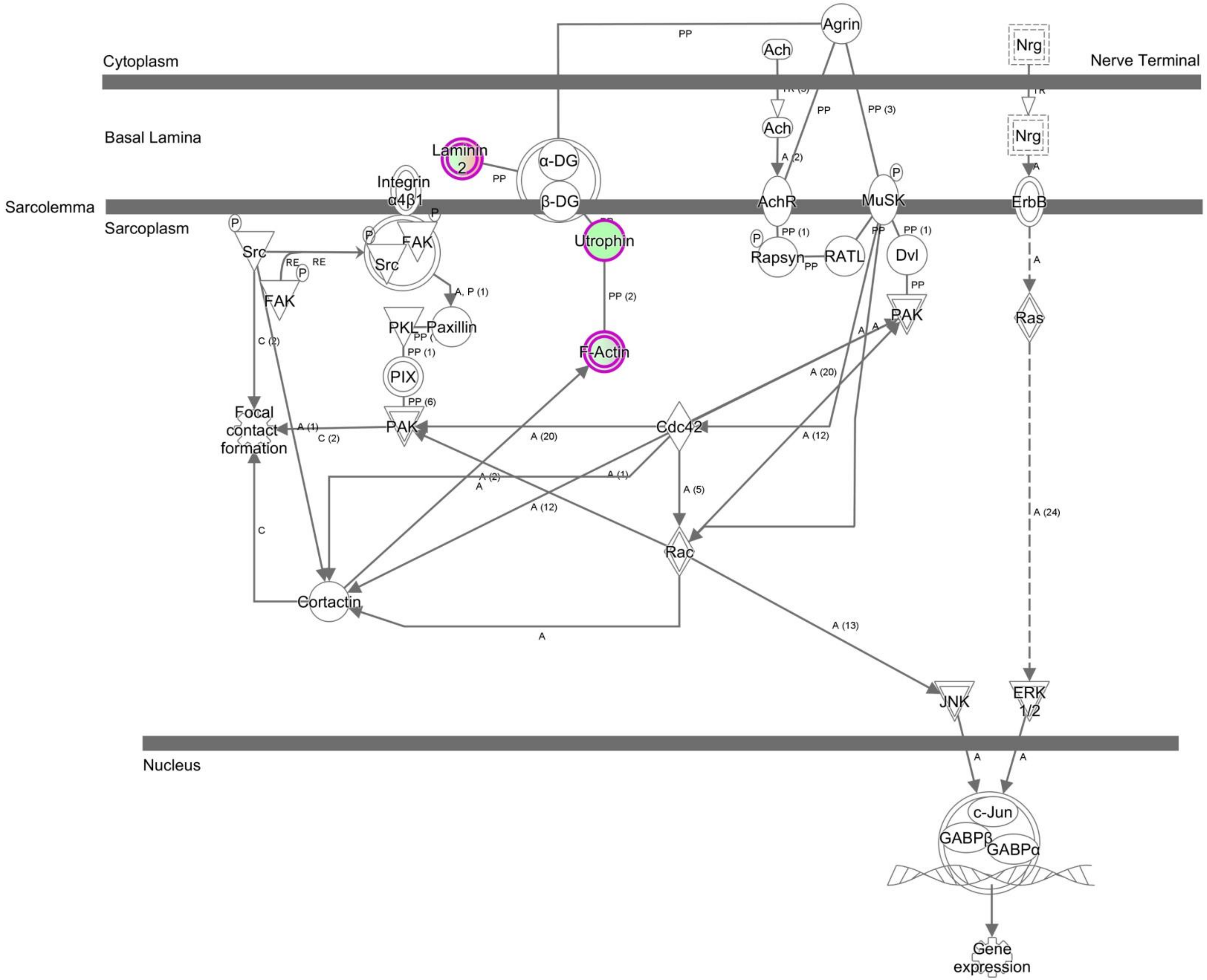

© 2000-2016 QIAGEN. All rights reserved.

Hematopoiesis from Pluripotent Stem Cells

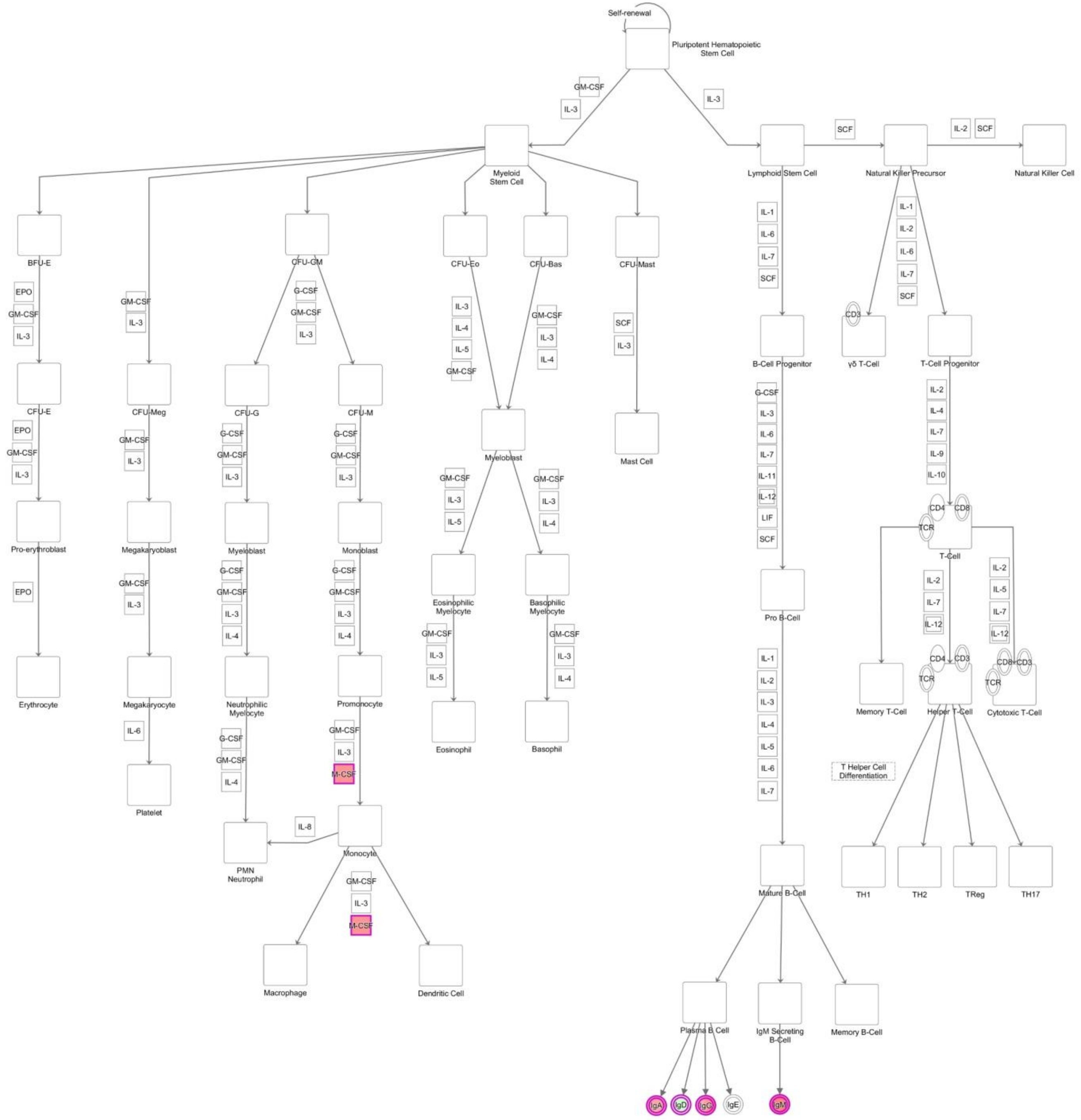

© 2000-2016 QIAGEN. All rights reserved.

PPMS

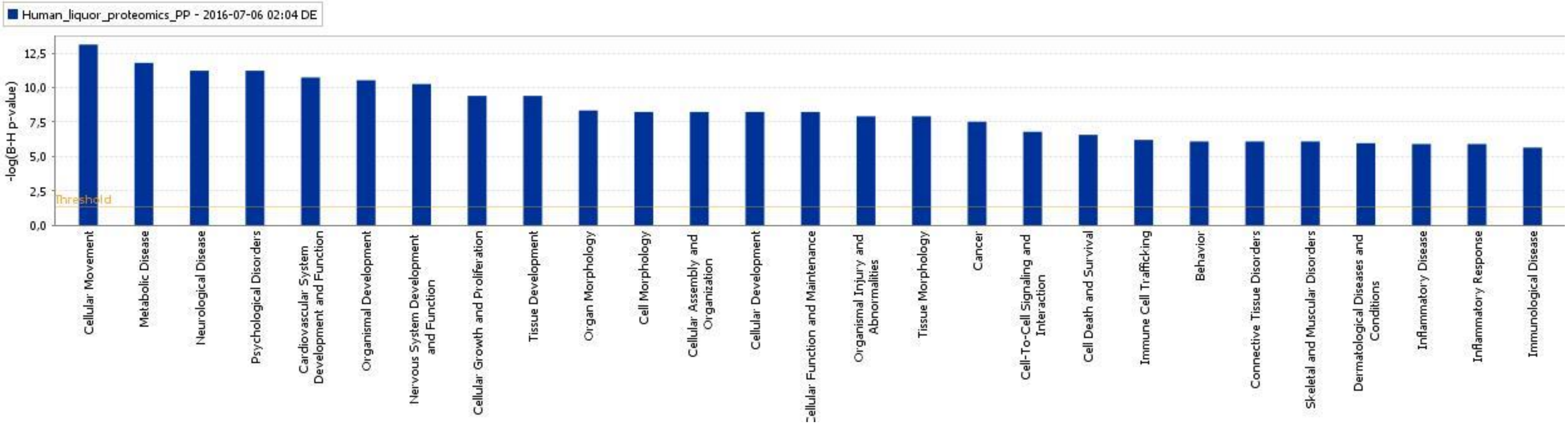
Human_liquor_proteomics_PP - 2016-07-06 02:04 DE
-log(B-H p-value)
12,5
10,0
7,5
5,0
2,5
0,0
Threshold
Cellular Movement
Metabolic Disease
Neurological Disease
Psychological Disorders
Cardiovascular System Development and Function
Organismal Development
Nervous System Development and Function
Cellular Growth and Proliferation
Tissue Development
Organ Morphology
Cell Morphology
Cellular Assembly and Organization
Cellular Development
Cellular Function and Maintenance
Organismal Injury and Abnormalities
Tissue Morphology
Cancer
Cell-To-Cell Signaling and Interaction
Cell Death and Survival
Immune Cell Trafficking
Behavior
Connective Tissue Disorders
Skeletal and Muscular Disorders
Dermatological Diseases and Conditions
Inflammatory Disease
Inflammatory Response
Immunological Disease

Analysis: Human_liquor_proteomics_PP - 2016-07-06 02:04 DE

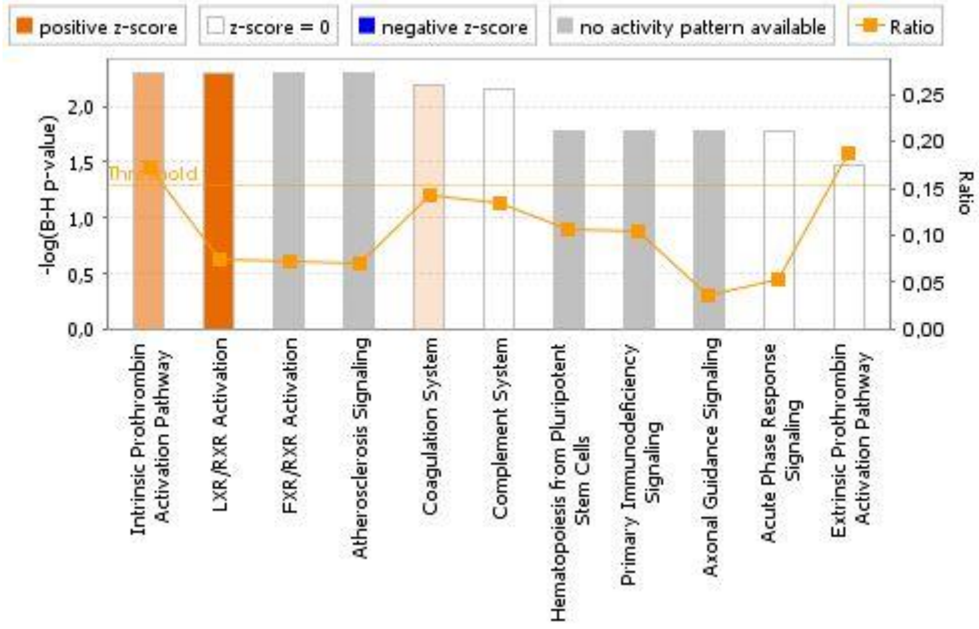

© 2000-2016 QIAGEN. All rights reserved.

Analysis: Human_liquor_proteomics_PP - 2016-07-06 02:04 DE

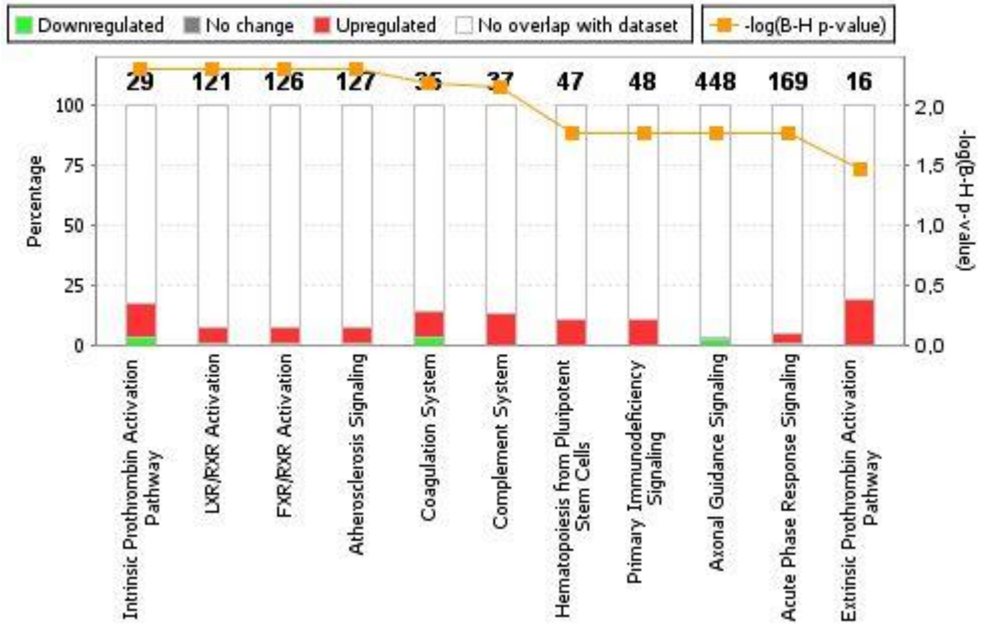

© 2000-2016 QIAGEN. All rights reserved.

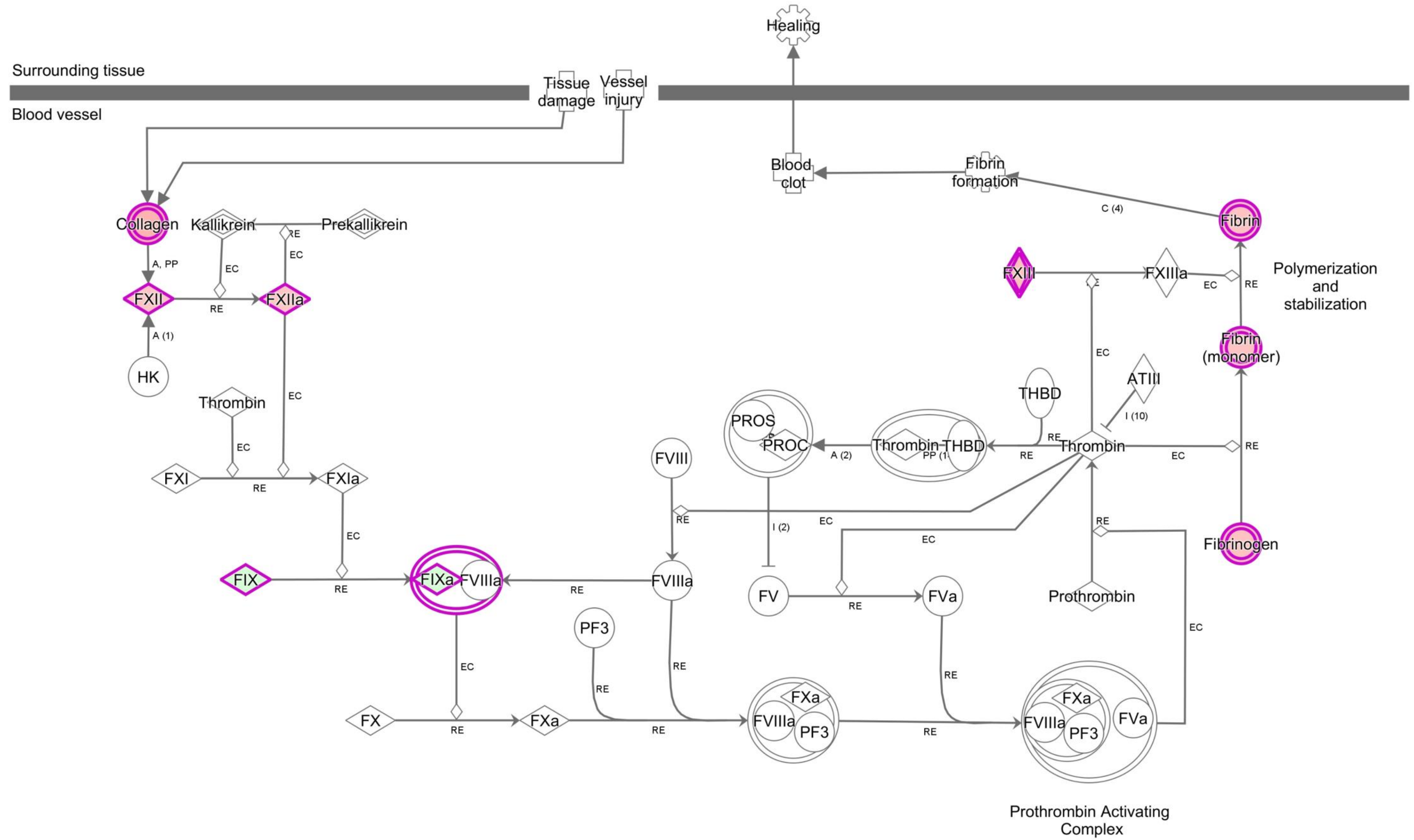

© 2000-2016 QIAGEN. All rights reserved.

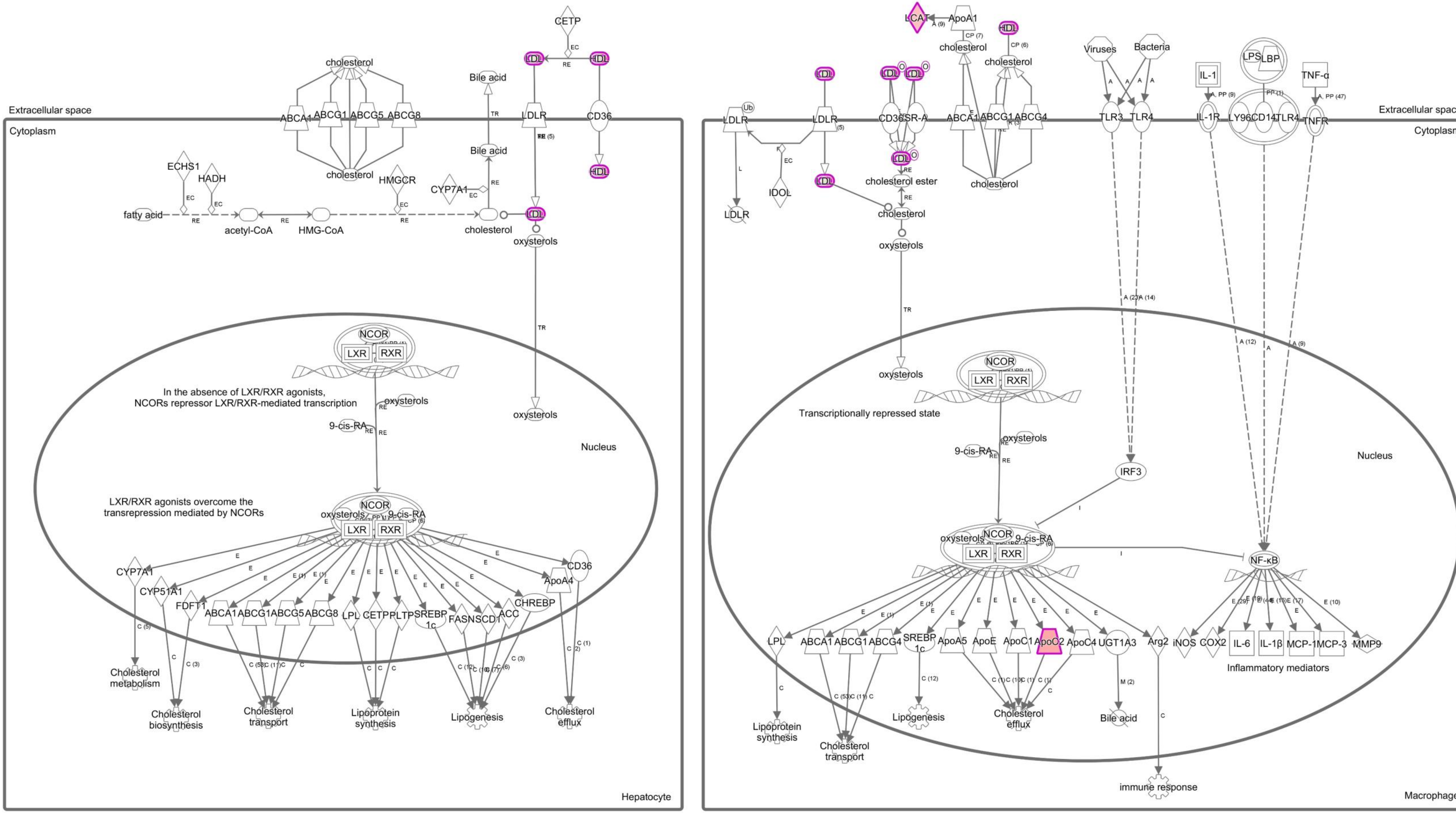

© 2000-2016 QIAGEN. All rights reserved.

FXR/RXR Activation

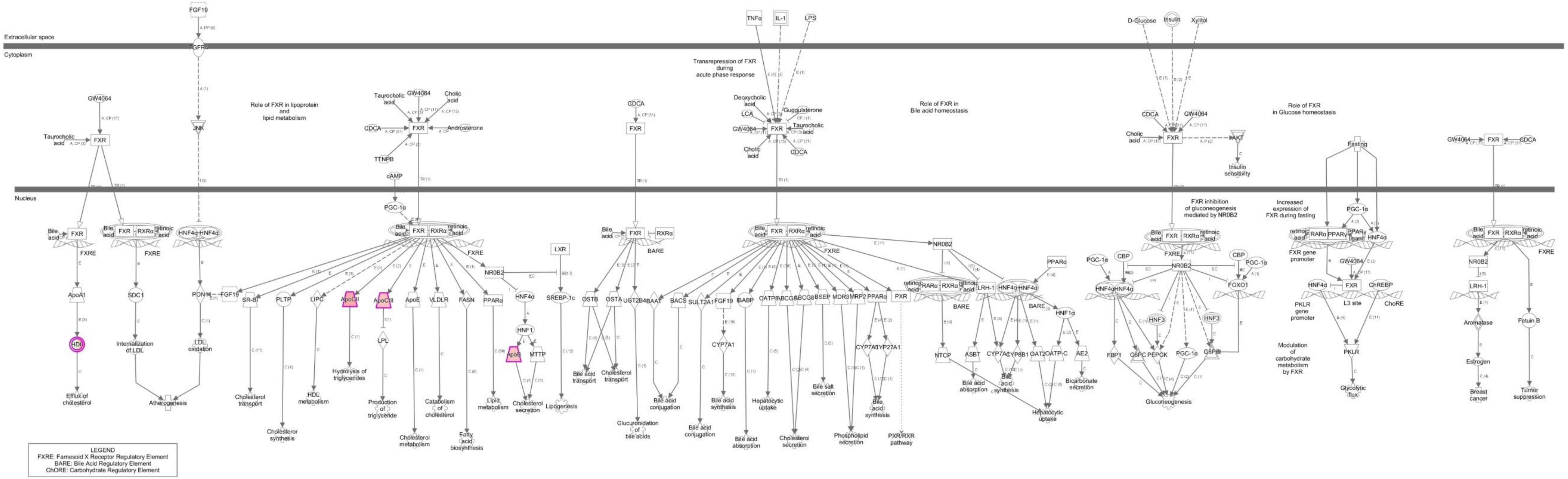

© 2000-2016 QIAGEN. All rights reserved.

Axonal Guidance Signaling

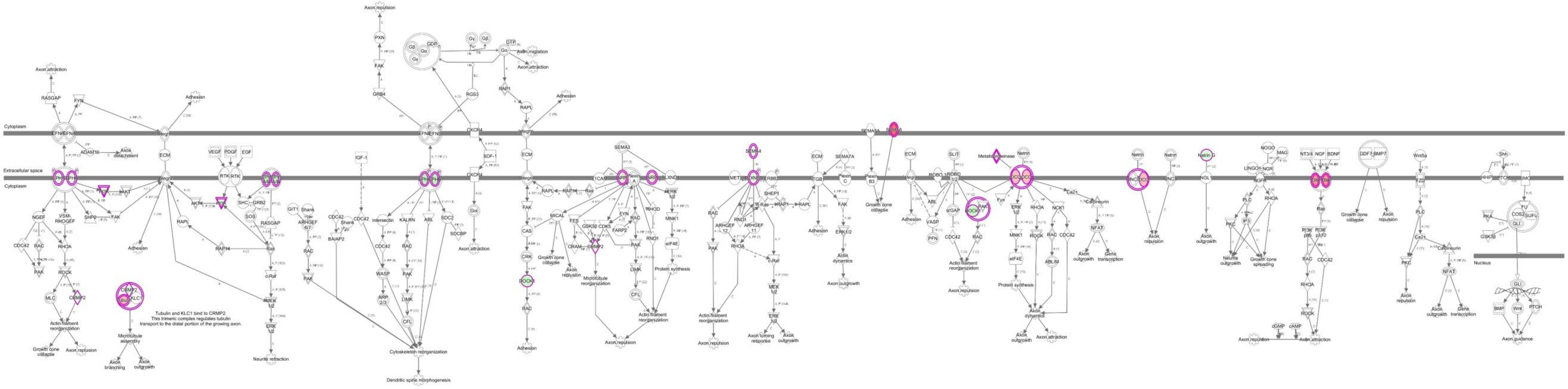

© 2000-2016 QIAGEN. All rights reserved.

SPMS

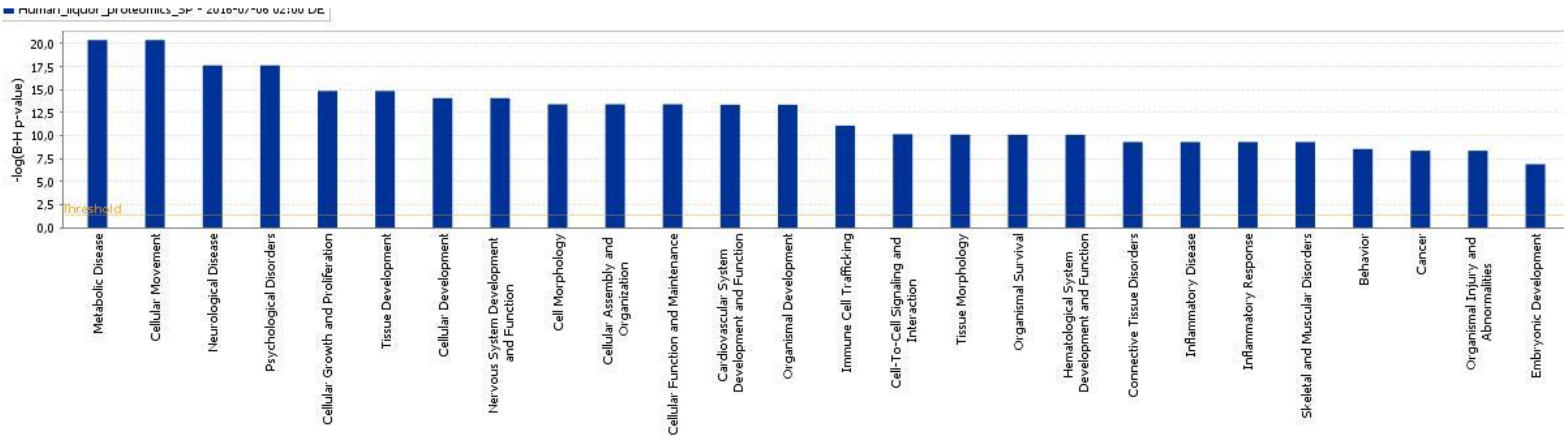

Human_liquor_proteomics_SP - 2016-07-06 02:00 DE
-log(B-H p-value)
20,0
17,5
15,0
12,5
10,0
7,5
5,0
2,5
0,0
Threshold
Metabolic Disease
Cellular Movement
Neurological Disease
Psychological Disorders
Cellular Growth and Proliferation
Tissue Development
Cellular Development
Nervous System Development and Function
Cell Morphology
Cellular Assembly and Organization
Cellular Function and Maintenance
Cardiovascular System Development and Function
Organismal Development
Immune Cell Trafficking
Cell-To-Cell Signaling and Interaction
Tissue Morphology
Organismal Survival
Hematological System Development and Function
Connective Tissue Disorders
Inflammatory Disease
Inflammatory Response
Skeletal and Muscular Disorders
Behavior
Cancer
Organismal Injury and Abnormalities
Embryonic Development

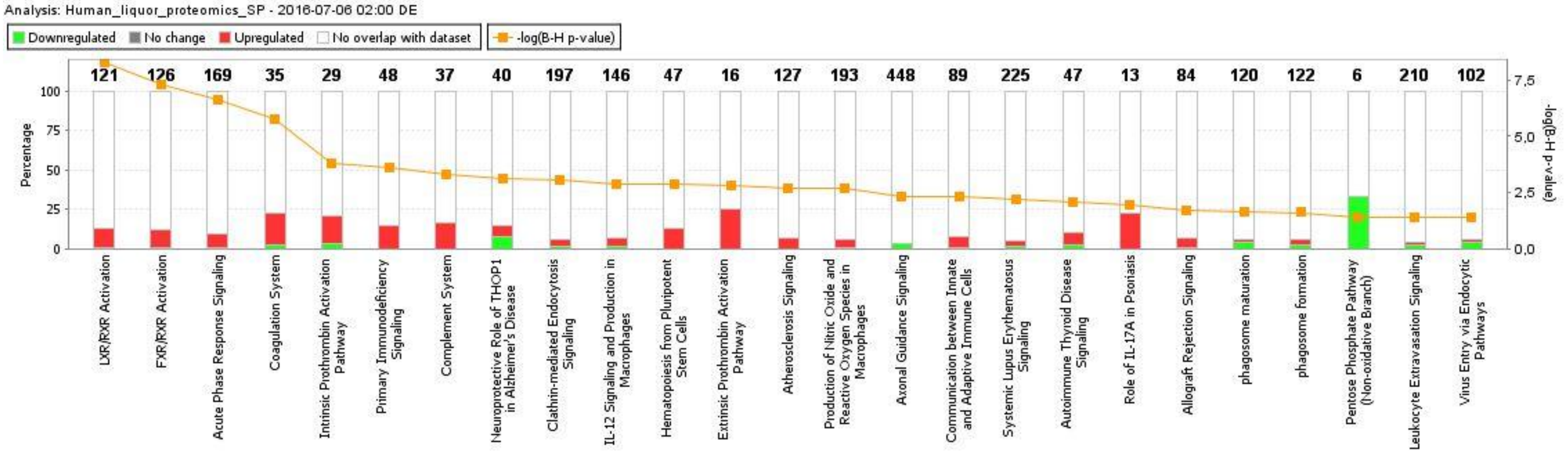

© 2000-2016 QIAGEN. All rights reserved.

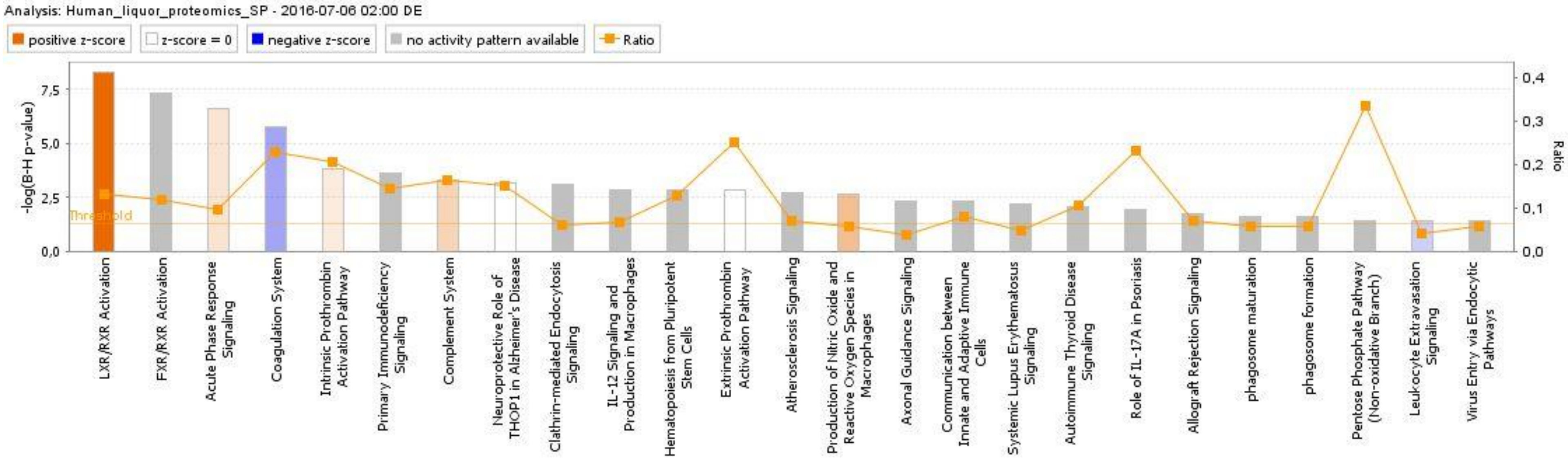

© 2000-2016 QIAGEN. All rights reserved.

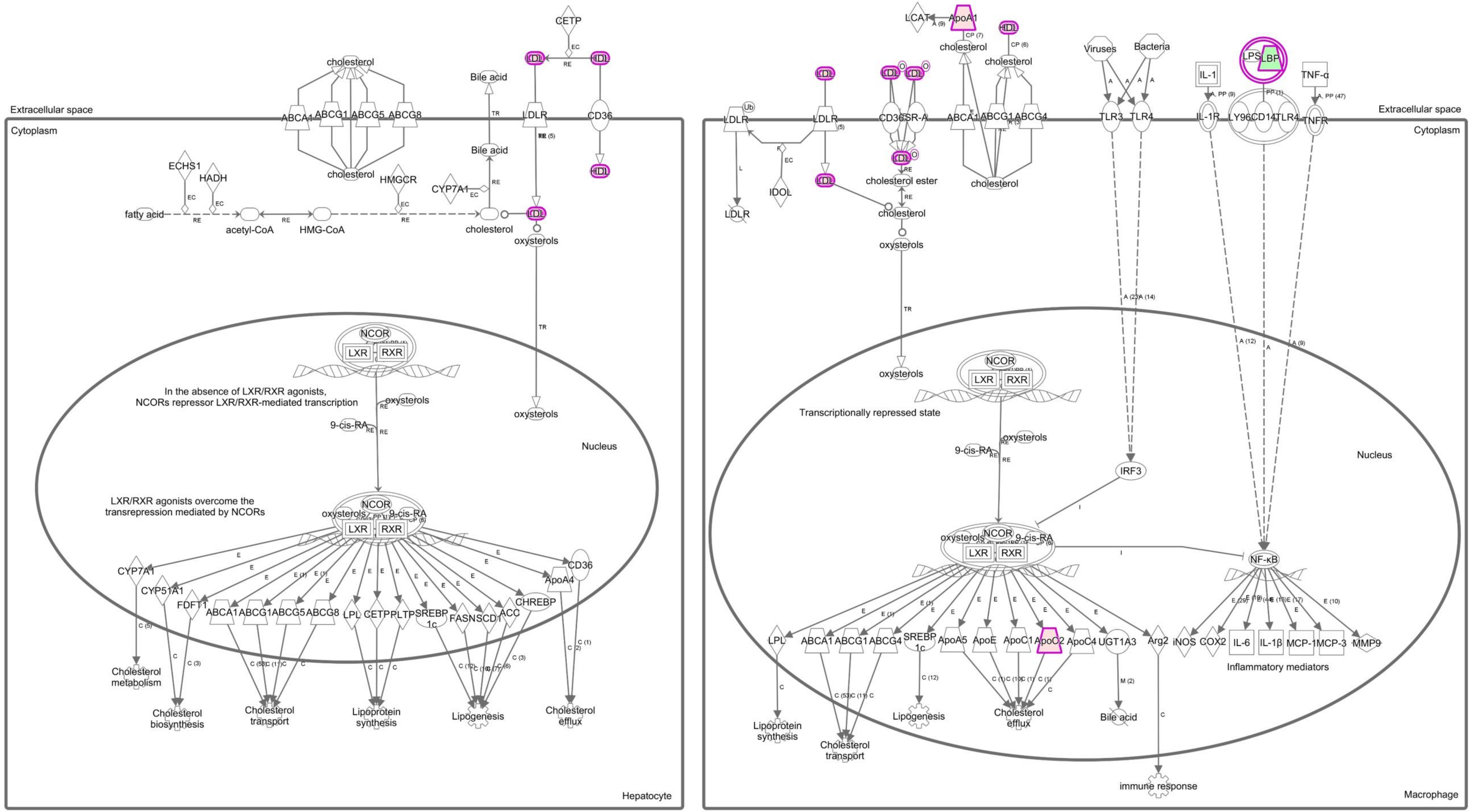

© 2000-2016 QIAGEN. All rights reserved.

Neuroprotective Role of THOP1 in Alzheimer's Disease

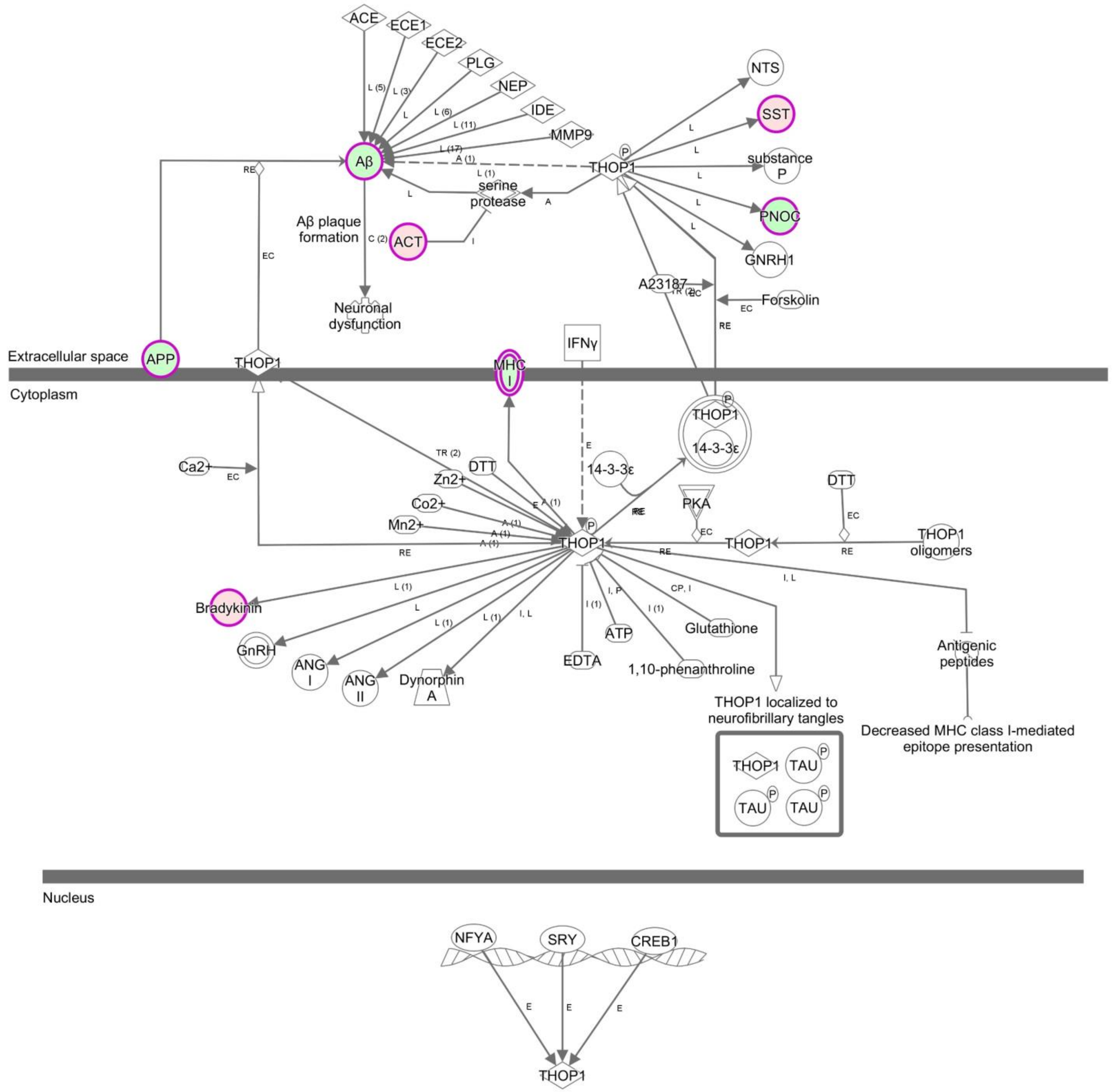

© 2000-2016 QIAGEN. All rights reserved.

Coagulation System

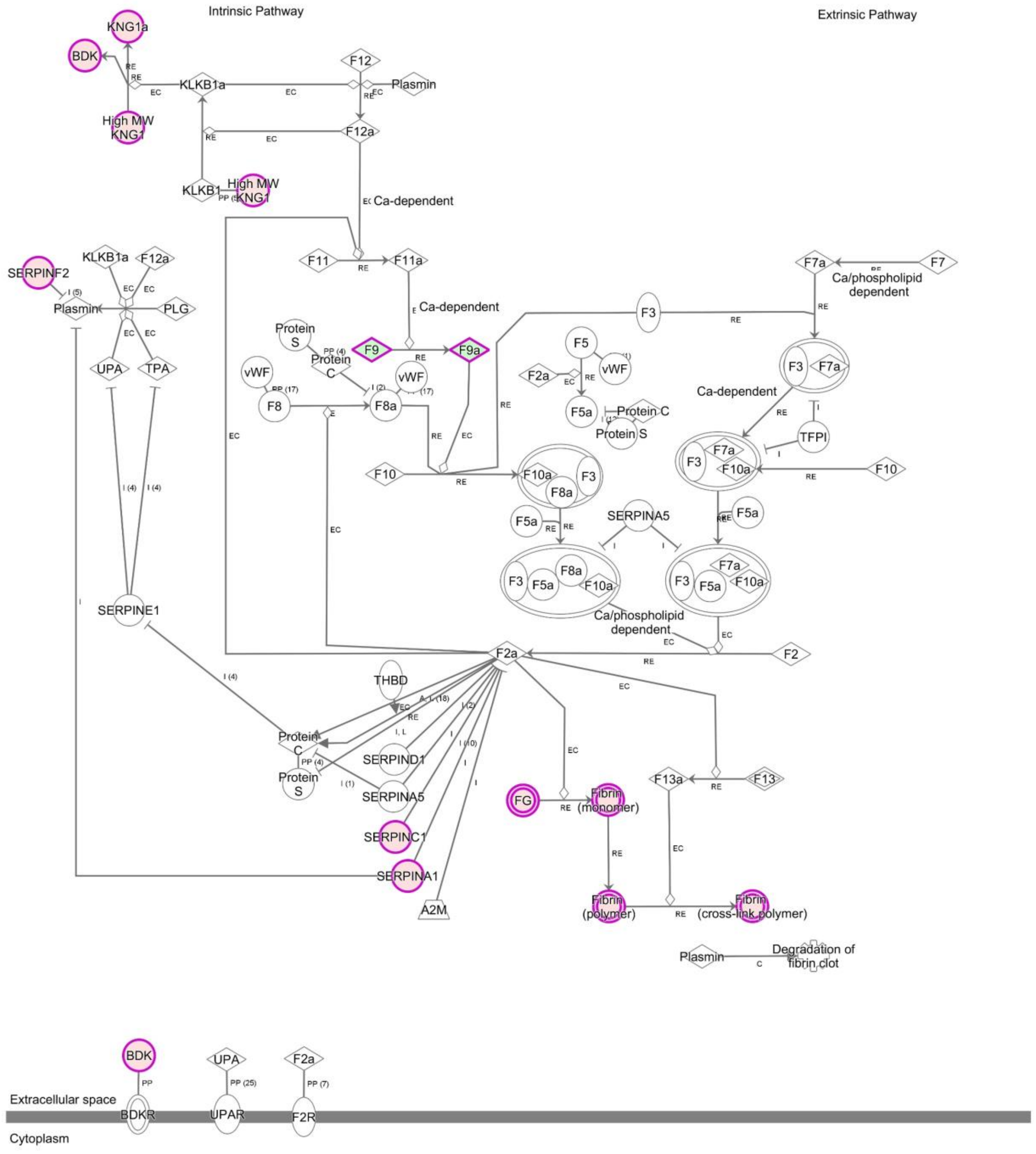

© 2000-2016 QIAGEN. All rights reserved.

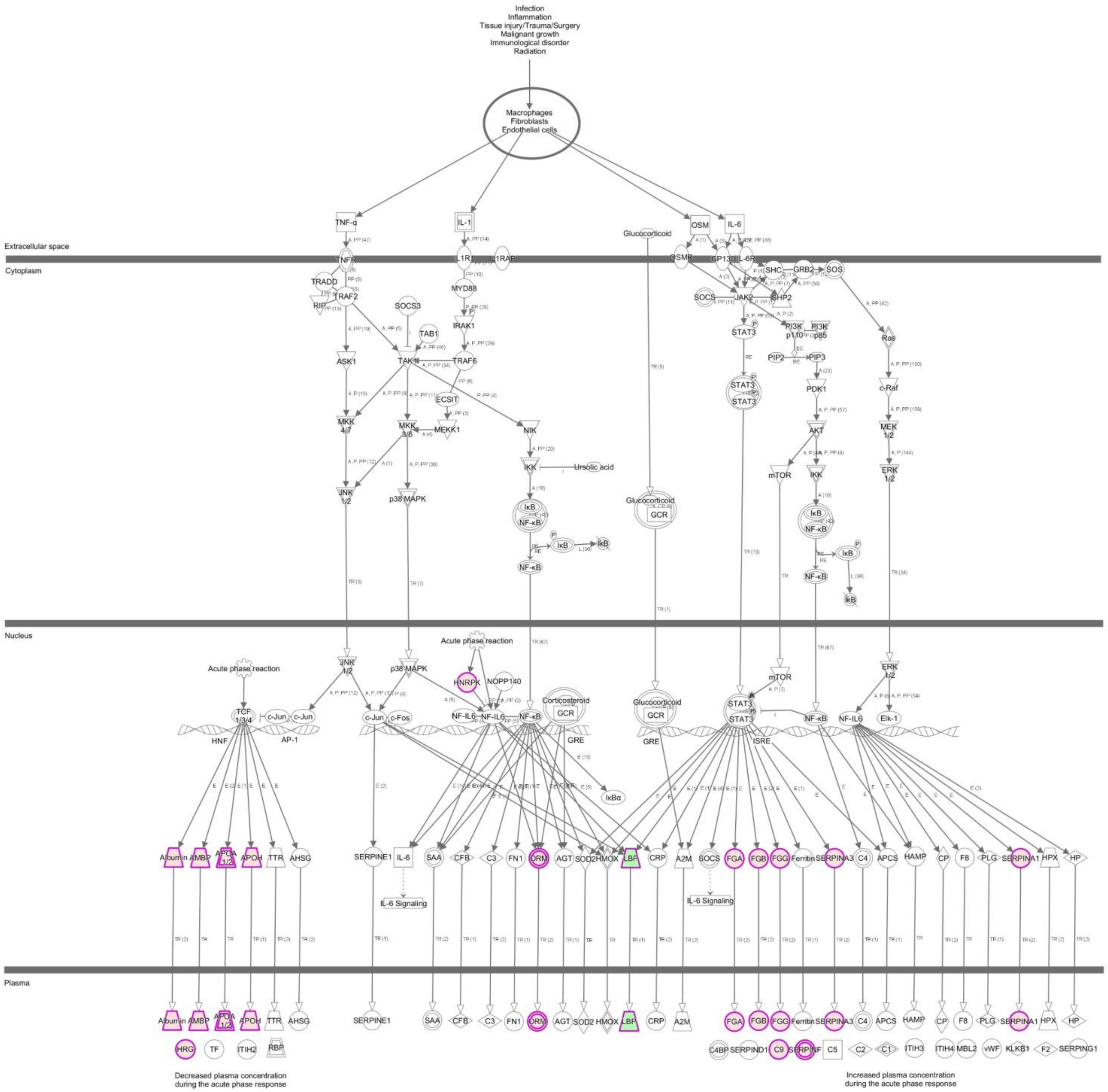

© 2000-2016 QIAGEN. All rights reserved.

AQP4-IgG$^+$ NMOSD

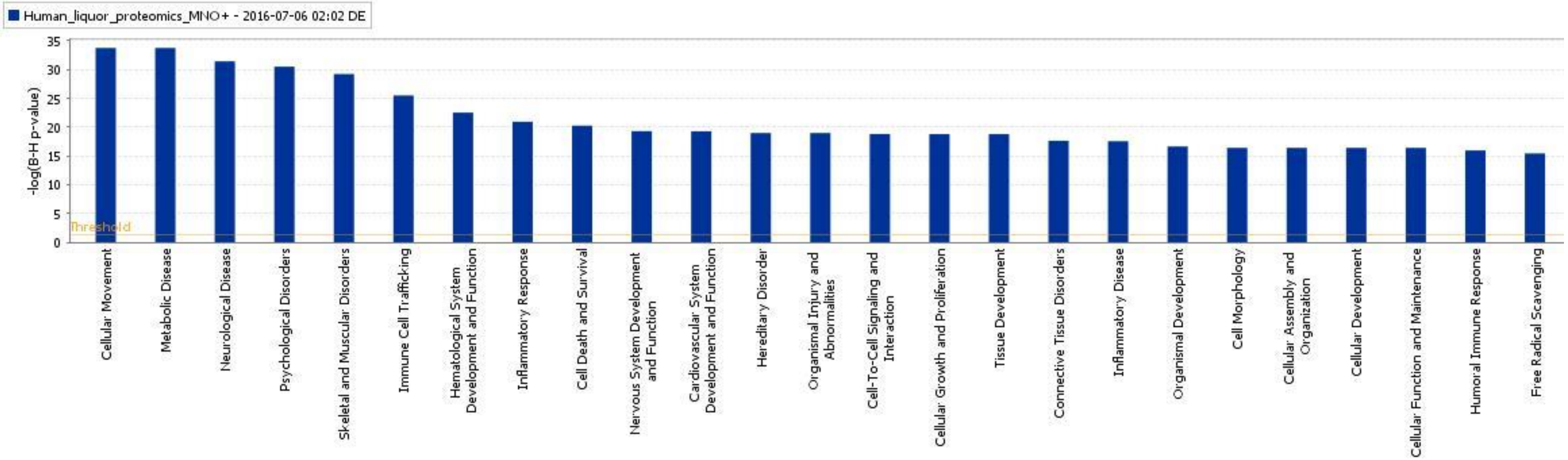
Human_liquor_proteomics_MNO+ - 2016-07-06 02:02 DE
-log(B-H p-value)
35
30
25
20
15
10
5
0
Threshold
Cellular Movement
Metabolic Disease
Neurological Disease
Psychological Disorders
Skeletal and Muscular Disorders
Immune Cell Trafficking
Hematological System Development and Function
Inflammatory Response
Cell Death and Survival
Nervous System Development and Function
Cardiovascular System Development and Function
Hereditary Disorder
Organismal Injury and Abnormalities
Cell-To-Cell Signaling and Interaction
Cellular Growth and Proliferation
Tissue Development
Connective Tissue Disorders
Inflammatory Disease
Organismal Development
Cell Morphology
Cellular Assembly and Organization
Cellular Development
Cellular Function and Maintenance
Humoral Immune Response
Free Radical Scavenging

Analysis: Human_liquor_proteomics_MNO+ - 2016-07-06 02:02 DE

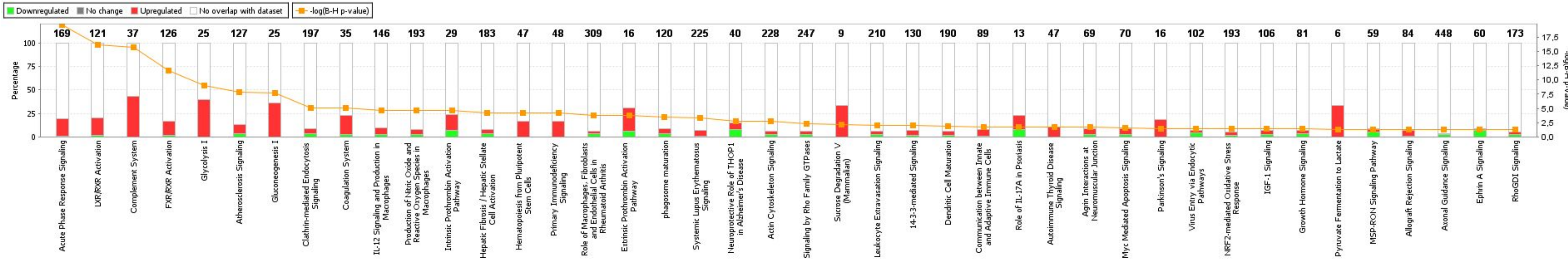

© 2000-2016 QIAGEN. All rights reserved.

Analysis: Human_liquor_proteomics_MNO+ - 2016-07-06 02:02 DE

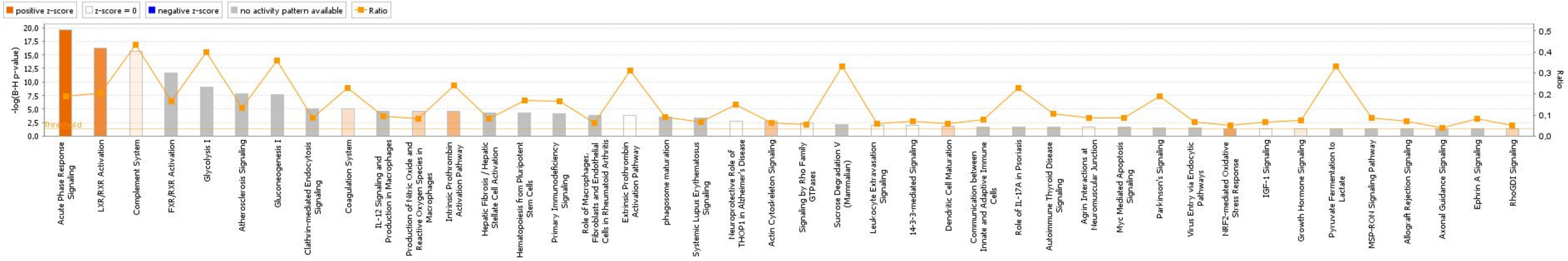

© 2000-2016 QIAGEN. All rights reserved.

LXR/RXR Activation

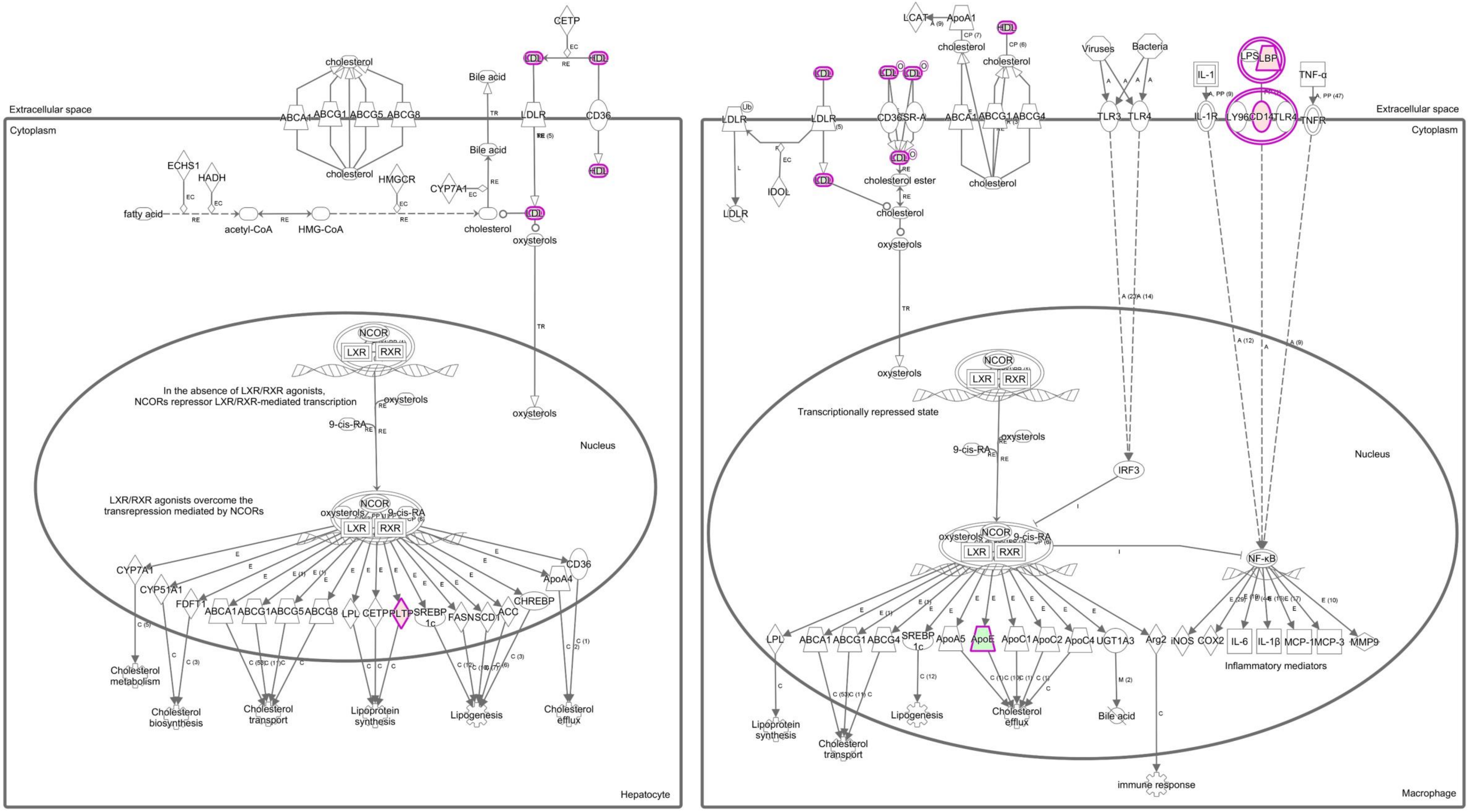

© 2000-2016 QIAGEN. All rights reserved.

Complement System

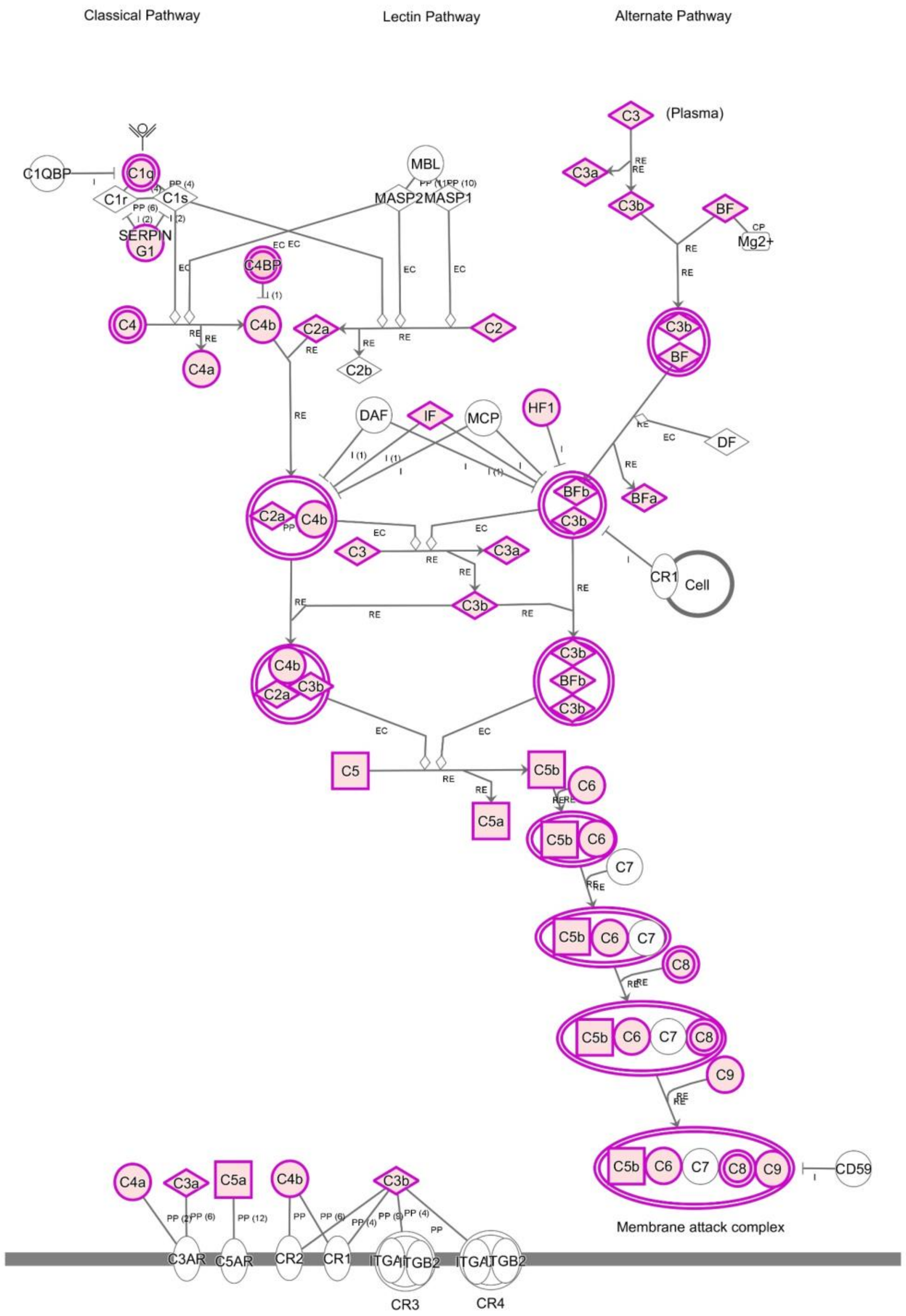

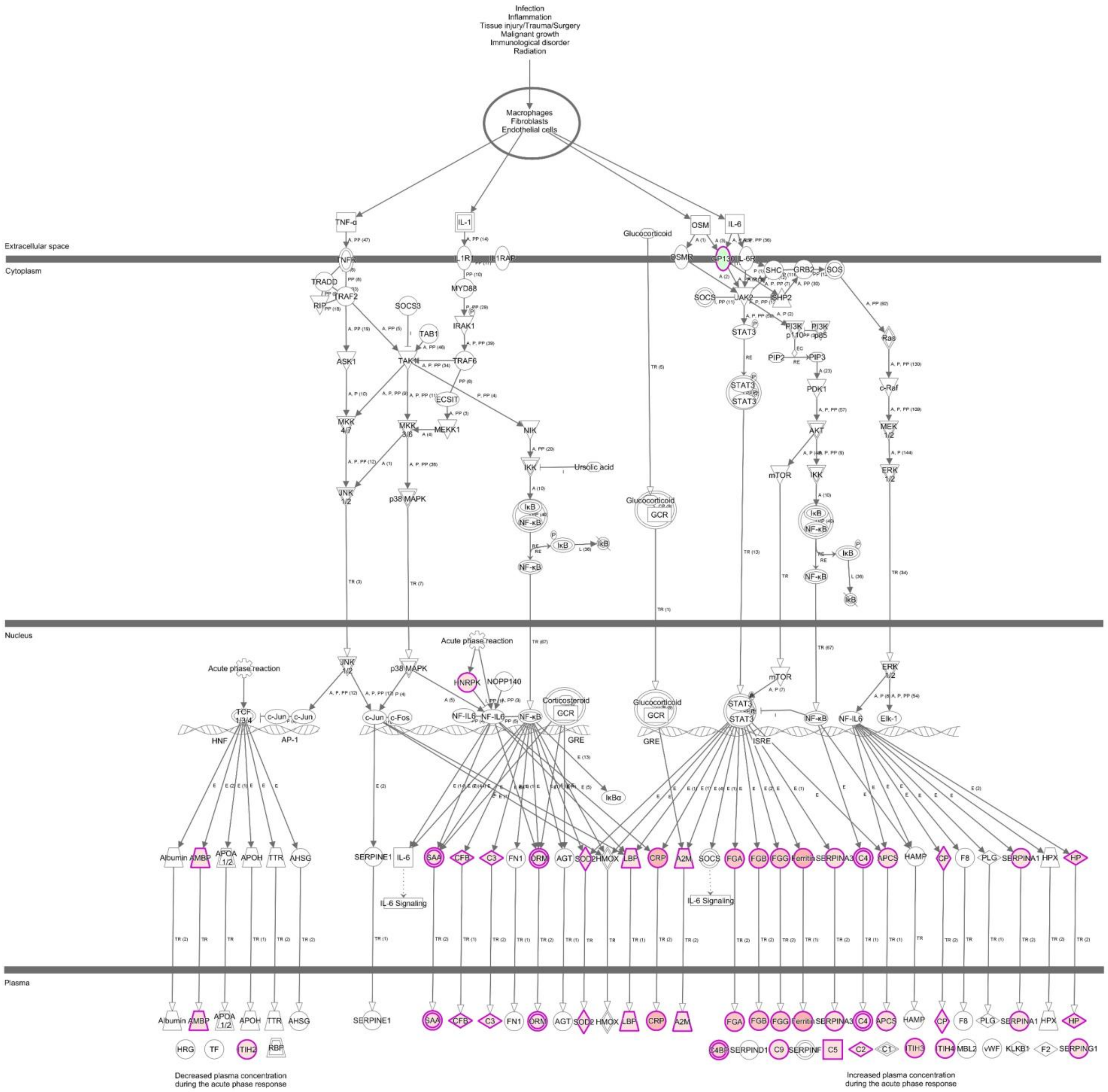

© 2000-2016 QIAGEN. All rights reserved.

AD

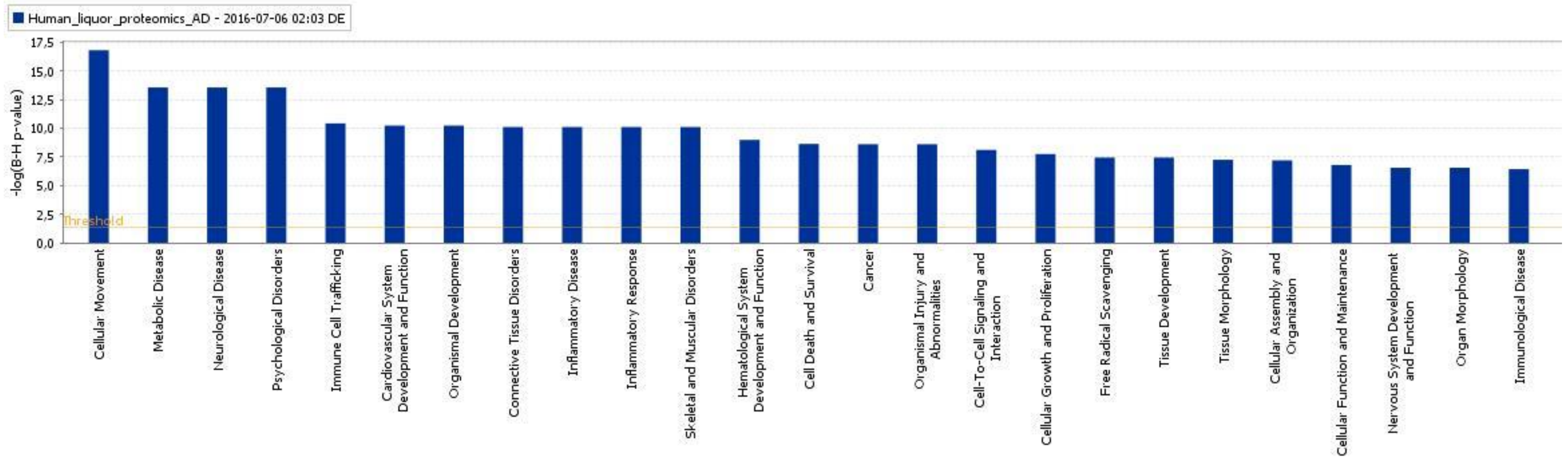

Human_liquor_proteomics_AD - 2016-07-06 02:03 DE
-log(B-H p-value)
17,5
15,0
12,5
10,0
7,5
5,0
2,5
0,0
Threshold
Cellular Movement
Metabolic Disease
Neurological Disease
Psychological Disorders
Immune Cell Trafficking
Cardiovascular System Development and Function
Organismal Development
Connective Tissue Disorders
Inflammatory Disease
Inflammatory Response
Skeletal and Muscular Disorders
Hematological System Development and Function
Cell Death and Survival
Cancer
Organismal Injury and Abnormalities
Cell-To-Cell Signaling and Interaction
Cellular Growth and Proliferation
Free Radical Scavenging
Tissue Development
Tissue Morphology
Cellular Assembly and Organization
Cellular Function and Maintenance
Nervous System Development and Function
Organ Morphology
Immunological Disease

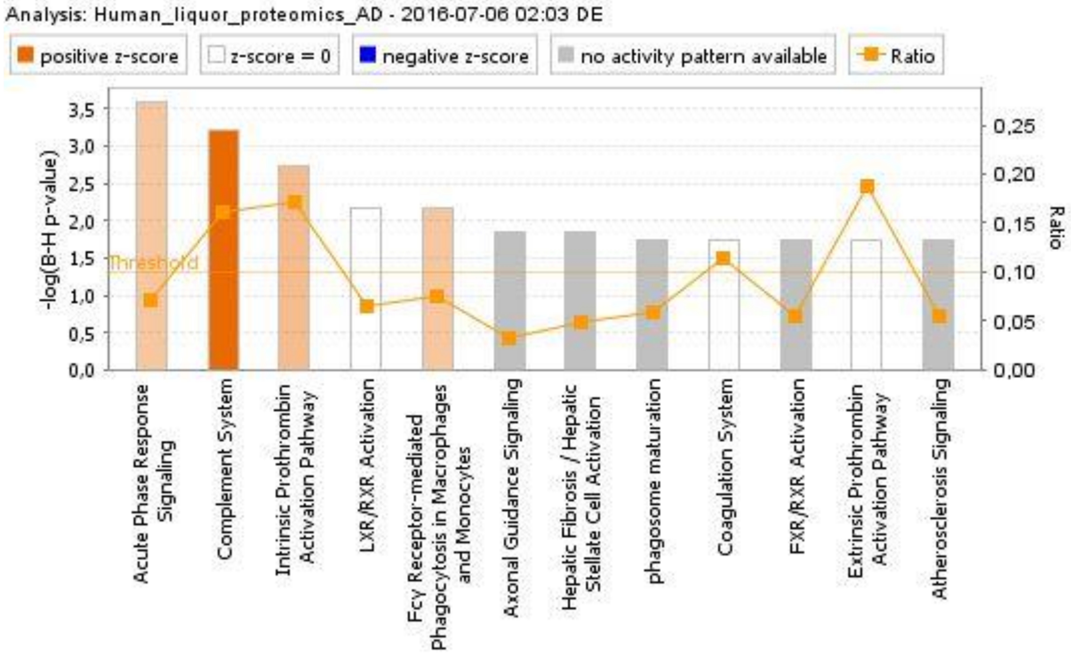

© 2000-2016 QIAGEN. All rights reserved.

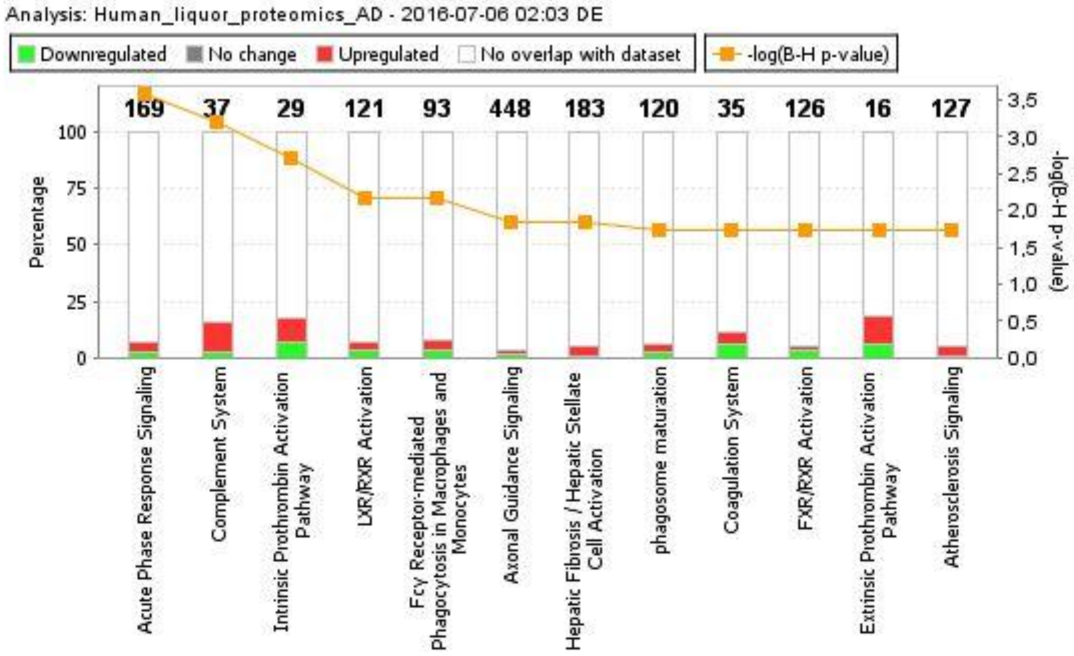

© 2000-2016 QIAGEN. All rights reserved.

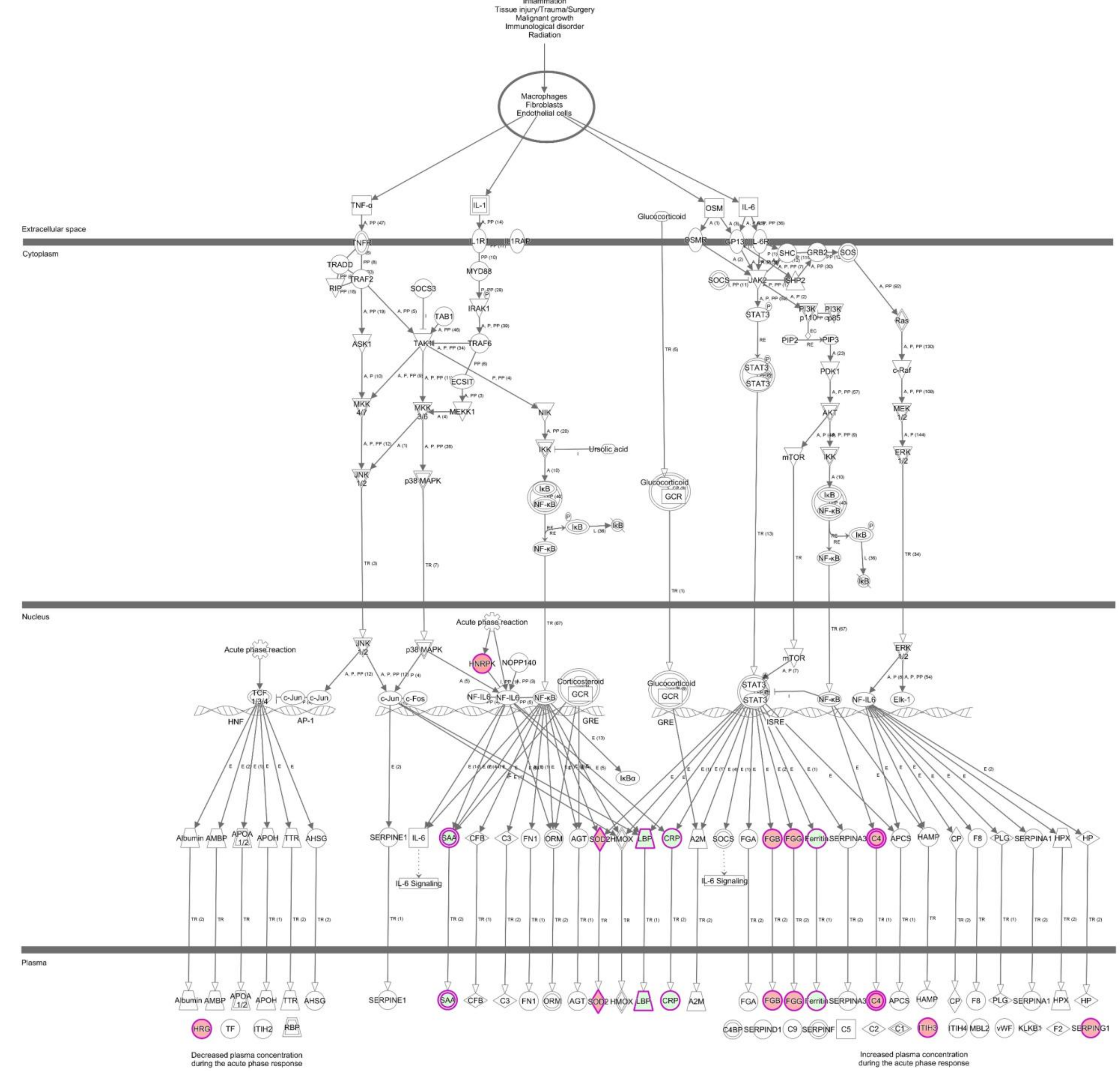

© 2000-2016 QIAGEN. All rights reserved.

Complement System

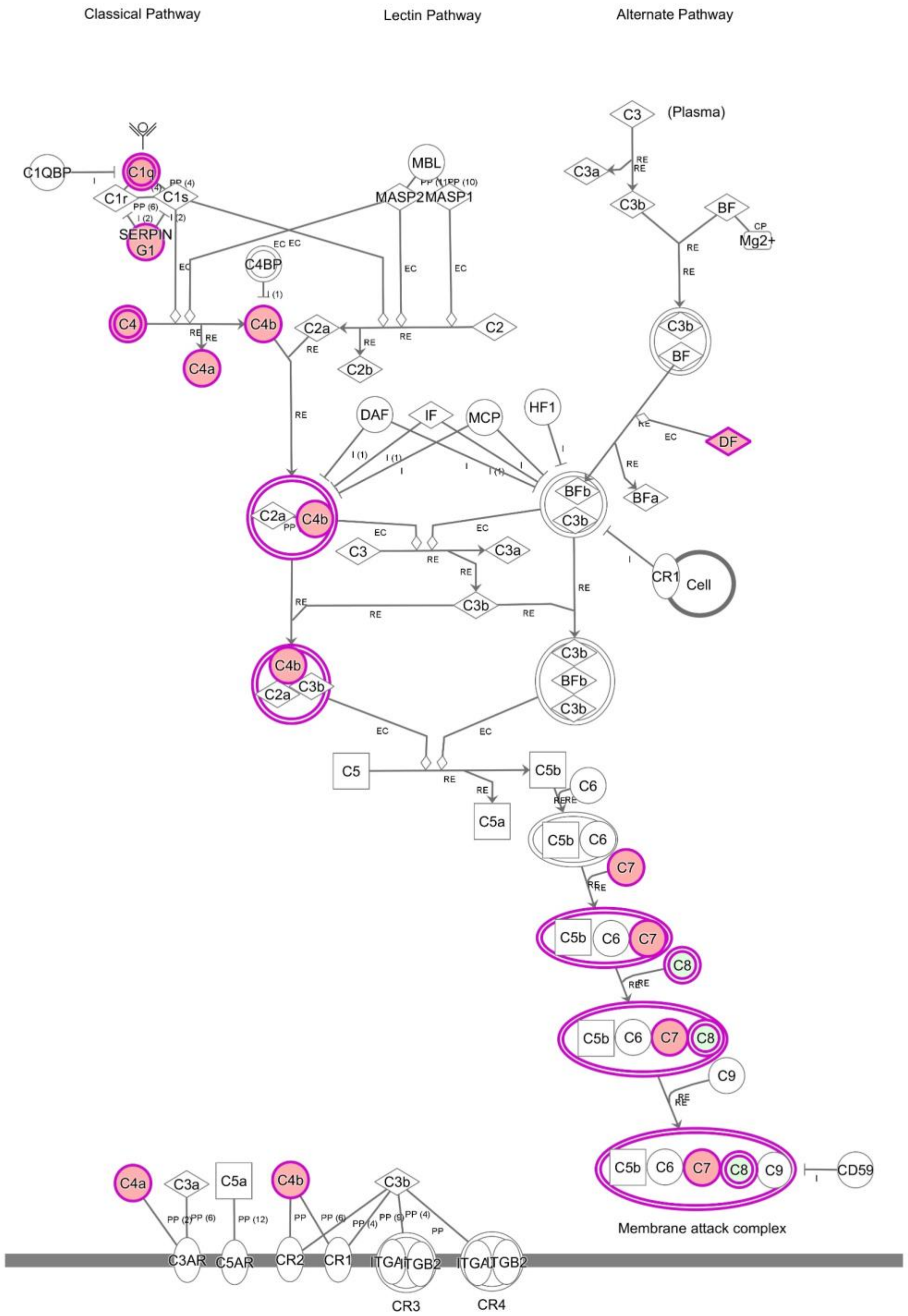

© 2000-2016 QIAGEN. All rights reserved.

Axonal Guidance Signaling

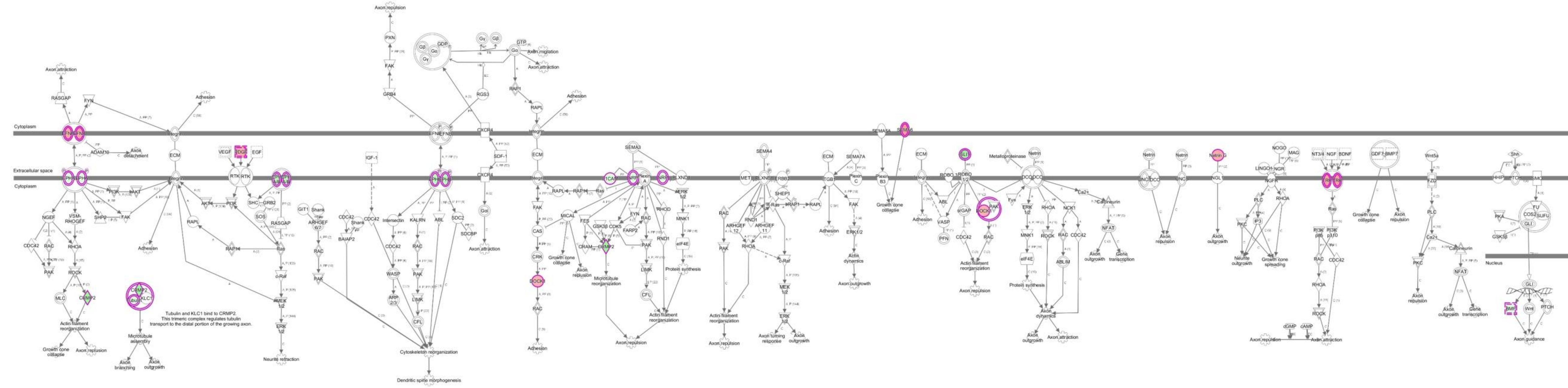

© 2000-2016 QIAGEN. All rights reserved.
