# Supplementary material for: CSF proteome in multiple sclerosis subtypes related to brain lesion transcriptomes
Source: Sci Rep. 2021 Feb 18;11:4132. doi: 10.1038/s41598-021-83591-5 (PMC7892884; doi:10.1038/s41598-021-83591-5)
Supplement: Supplementary file 5 — Supplementary Table S2. [file 41598_2021_83591_MOESM5_ESM.docx]

**Supplementary Table S2**

The 299 proteins selected from the three different statistical tests for quantification in individual CSF samples

| Statistical tests | Protein no. | Protein ID |
| --- | --- | --- |
| ANOVA, Complementary analysis, limma | 2 | P36222 |
|  |  | P01033 |
| ANOVA, limma | 112 | P02656 |
|  |  | Q92876 |
|  |  | P05155 |
|  |  | P01019 |
|  |  | Q9P0K1 |
|  |  | Q13449 |
|  |  | O14773 |
|  |  | Q96GW7 |
|  |  | P12109 |
|  |  | O00451 |
|  |  | Q14515 |
|  |  | P16870 |
|  |  | P02647 |
|  |  | P08123 |
|  |  | Q9P2S2 |
|  |  | P10645 |
|  |  | Q13228 |
|  |  | Q92765 |
|  |  | P02649 |
|  |  | Q96KN2 |
|  |  | P01210 |
|  |  | Q9BY67 |
|  |  | P01034 |
|  |  | P01023 |
|  |  | P47972 |
|  |  | Q9UBP4 |
|  |  | Q8NBJ4 |
|  |  | Q9Y5Y7 |
|  |  | P35908 |
|  |  | O00533 |
|  |  | P05452 |
|  |  | Q99435 |
|  |  | Q96FE7 |
|  |  | P00558 |
|  |  | P01011 |
|  |  | Q9BZR6 |
|  |  | Q8N126 |
|  |  | P05060 |
|  |  | O00391 |
|  |  | P13521 |
|  |  | Q9UBQ6 |
|  |  | P19021 |
|  |  | Q13740 |
|  |  | P43251 |
|  |  | P51693 |
|  |  | Q06481 |
|  |  | Q8N3J6 |
|  |  | P23470 |
|  |  | P55290 |
|  |  | P02750 |
|  |  | P54764 |
|  |  | O43505 |
|  |  | Q9Y2T3 |
|  |  | Q8WXD2 |
|  |  | P05090 |
|  |  | P78324 |
|  |  | P55285 |
|  |  | Q99574 |
|  |  | Q8NFZ8 |
|  |  | Q92823 |
|  |  | P49641 |
|  |  | O94772 |
|  |  | P13591 |
|  |  | P61769 |
|  |  | P27169 |
|  |  | P23142 |
|  |  | O94985 |
|  |  | P19827 |
|  |  | P05067 |
|  |  | P04075 |
|  |  | P02743 |
|  |  | Q15818 |
|  |  | P78509 |
|  |  | Q15166 |
|  |  | P02655 |
|  |  | P25311 |
|  |  | O15240 |
|  |  | P02787 |
|  |  | P35858 |
|  |  | P07602 |
|  |  | P43121 |
|  |  | P04156 |
|  |  | P18428 |
|  |  | Q12860 |
|  |  | P10451 |
|  |  | P45877 |
|  |  | Q14118 |
|  |  | Q02246 |
|  |  | P04216 |
|  |  | Q9NRN5 |
|  |  | P13473 |
|  |  | Q9NQ79 |
|  |  | P07195 |
|  |  | Q9UHG2 |
|  |  | P05408 |
|  |  | Q53EL9 |
|  |  | P02763 |
|  |  | P27797 |
|  |  | Q9P121 |
|  |  | Q9UM22 |
|  |  | P48058 |
|  |  | O95502 |
|  |  | Q9Y4C0 |
|  |  | Q9ULB1 |
|  |  | Q92520 |
|  |  | Q15904 |
|  |  | Q6UX73 |
|  |  | Q86VB7 |
|  |  | Q14982 |
|  |  | P04114 |
|  |  | P54289 |
|  |  | O95196 |
| Complementary analysis, limma | 1 | P62258 |
| ANOVA | 34 | P02675 |
|  |  | Q13822 |
|  |  | Q96S96 |
|  |  | P01008 |
|  |  | O75509 |
|  |  | P02671 |
|  |  | P07339 |
|  |  | O75326 |
|  |  | Q16270 |
|  |  | P29622 |
|  |  | P07737 |
|  |  | P04003 |
|  |  | P06727 |
|  |  | Q9C0A0 |
|  |  | P48745 |
|  |  | P02765 |
|  |  | Q9Y646 |
|  |  | Q16610 |
|  |  | P02790 |
|  |  | P17900 |
|  |  | P06396 |
|  |  | Q92954 |
|  |  | P10909 |
|  |  | P00747 |
|  |  | Q14624 |
|  |  | P02753 |
|  |  | Q02818 |
|  |  | Q99674 |
|  |  | P41222 |
|  |  | P09486 |
|  |  | P20774 |
|  |  | P61916 |
|  |  | P69905 |
|  |  | Q6UXB8 |
| limma | 124 | Q06033 |
|  |  | P35052 |
|  |  | P20472 |
|  |  | P01042 |
|  |  | O94856 |
|  |  | O15031 |
|  |  | Q8WVQ1 |
|  |  | P19823 |
|  |  | Q5VST9 |
|  |  | P14618 |
|  |  | Q16568 |
|  |  | P33908 |
|  |  | P10599 |
|  |  | P00748 |
|  |  | P02774 |
|  |  | P00450 |
|  |  | Q9BUJ0 |
|  |  | P02751 |
|  |  | P23471 |
|  |  | P06276 |
|  |  | P32119 |
|  |  | P11362 |
|  |  | P13987 |
|  |  | O14594 |
|  |  | P09972 |
|  |  | P00441 |
|  |  | P17174 |
|  |  | Q8TAG5 |
|  |  | Q93091 |
|  |  | P21246 |
|  |  | P02452 |
|  |  | Q9NQX5 |
|  |  | P51801 |
|  |  | Q9NYQ8 |
|  |  | Q9UBX1 |
|  |  | O94769 |
|  |  | Q86UD1 |
|  |  | P62937 |
|  |  | Q7Z7M0 |
|  |  | Q9P202 |
|  |  | P11279 |
|  |  | P99999 |
|  |  | P02786 |
|  |  | Q9BXJ0 |
|  |  | Q92563 |
|  |  | Q6UWP8 |
|  |  | Q9NZP8 |
|  |  | P21802 |
|  |  | P32004 |
|  |  | Q969P0 |
|  |  | O94919 |
|  |  | P07477 |
|  |  | P02652 |
|  |  | O15394 |
|  |  | P61626 |
|  |  | P40925 |
|  |  | O60883 |
|  |  | P98160 |
|  |  | P08174 |
|  |  | P08185 |
|  |  | O14498 |
|  |  | Q9UHI8 |
|  |  | Q9UJJ9 |
|  |  | P05154 |
|  |  | P04217 |
|  |  | P48740 |
|  |  | Q13519 |
|  |  | P04004 |
|  |  | Q92859 |
|  |  | B9A064 |
|  |  | P31151 |
|  |  | Q9H1Z8 |
|  |  | Q9HAR2 |
|  |  | P18065 |
|  |  | P54756 |
|  |  | Q7Z3B1 |
|  |  | P07711 |
|  |  | Q6EMK4 |
|  |  | P06733 |
|  |  | P02654 |
|  |  | Q9BYH1 |
|  |  | P01344 |
|  |  | Q12805 |
|  |  | P23284 |
|  |  | Q6UW01 |
|  |  | P40189 |
|  |  | O43493 |
|  |  | P30086 |
|  |  | P55058 |
|  |  | Q9UBX5 |
|  |  | P09668 |
|  |  | P14136 |
|  |  | Q6UX71 |
|  |  | P22792 |
|  |  | O76096 |
|  |  | Q16769 |
|  |  | Q8NI35 |
|  |  | Q92743 |
|  |  | P01009 |
|  |  | P02746 |
|  |  | P14314 |
|  |  | P09382 |
|  |  | O94910 |
|  |  | Q13231 |
|  |  | Q99497 |
|  |  | O76061 |
|  |  | Q03167 |
|  |  | P35542 |
|  |  | Q15582 |
|  |  | P20933 |
|  |  | P09603 |
|  |  | P08571 |
|  |  | Q9UHL4 |
|  |  | Q96IY4 |
|  |  | Q8IWU5 |
|  |  | P19652 |
|  |  | P14543 |
|  |  | Q13332 |
|  |  | P05546 |
|  |  | P26992 |
|  |  | P07333 |
|  |  | O60888 |
|  |  | P04196 |
|  |  | A6NLU5 |
| Complementary analysis | 26 | P02461 |
|  |  | Q13867 |
|  |  | P03952 |
|  |  | P30041 |
|  |  | P63104 |
|  |  | Q13634 |
|  |  | Q6ZMI3 |
|  |  | Q08345 |
|  |  | Q6UXD5 |
|  |  | P04271 |
|  |  | P08670 |
|  |  | Q9BTY2 |
|  |  | P25713 |
|  |  | P81605 |
|  |  | P14151 |
|  |  | Q9UKR8 |
|  |  | P00568 |
|  |  | P04080 |
|  |  | P13796 |
|  |  | O00468 |
|  |  | P80188 |
|  |  | P55786 |
|  |  | P60709 |
|  |  | P00915 |
|  |  | P05109 |
|  |  | P80108 |
